# Peptide nucleic acids (PNAs) control function of SARS-CoV-2 frameshifting stimulatory element trough PNA-RNA-PNA triplex formation

**DOI:** 10.1016/j.heliyon.2024.e33914

**Published:** 2024-06-29

**Authors:** Md Motiar Rahman, Christopher A. Ryan, Brandon R. Tessier, Eriks Rozners

**Affiliations:** Department of Chemistry, Binghamton University, The State University of New York, Binghamton, NY, 13902, United States

## Abstract

The highly structured nature of the SARS-CoV-2 genome provides many promising antiviral drug targets. One particularly promising target is a *cis*-acting RNA pseudoknot found within a critical region called the frameshifting stimulatory element (FSE). In this study, peptide nucleic acids (PNAs) binding to stem 2 of FSE RNA inhibited protein translation and frameshifting, as measured by a cell-free dual luciferase assay, more effectively than PNAs binding to stem 1, stem 3, or the slippery site. Surprisingly, simple antisense PNAs were stronger disruptors of frameshifting than PNA tail-clamps, despite higher thermal stability of the PNA-RNA-PNA triplexes formed by the latter. Another unexpected result was a strong and sequence non-specific enhancement of frameshifting inhibition when using a cationic triplex-forming PNA in conjunction with an antisense PNA targeting key regions of the frameshifting element. Our results illustrate both the potential and the challenges of using antisense PNAs to target highly structured RNAs, such as SARS-CoV-2 pseudoknots. While triplex forming PNAs, including PNA tail-clamps, are emerging as promising ligands for RNA recognition, the binding affinity enhancements when using cationic modifications in triplex-forming PNAs must be carefully balanced to avoid loss of sequence specificity in complex biological systems.

## Highlights

• Binding of peptide nucleic acids (PNAs) inhibited protein translation and frameshifting of SARS-CoV-2 RNA.
• Surprisingly, duplex-forming antisense PNAs were stronger inhibitors of frameshifting than triplex-forming PNA tail-clamps.
• Frameshifting inhibition did not correlate with PNA affinity and PNA-RNA complex thermal stability.
• Sequence non-specific inhibition of frameshifting was observed for cationic triplex-forming PNAs.

## 1 Introduction

SARS-CoV-2 is a positive-strand RNA virus belonging to the family Coronaviridae [1,2]. The single-stranded RNA ((+) ssRNA) genome of SARS-CoV-2 serves as a template for replication, transcription, and translation of viral proteins [1,2]. The diverse and critical mechanisms by which coronaviruses control replication and translation are not completely understood. However, highly conserved structural motifs, such as hairpins and pseudoknots, in the 5′ and 3′ untranslated regions (UTRs) and the protein-encoding regions of the viral RNA are critical for viral replication and mRNA transcription and translation [[3], [4], [5], [6]].

**Fig. 1:**
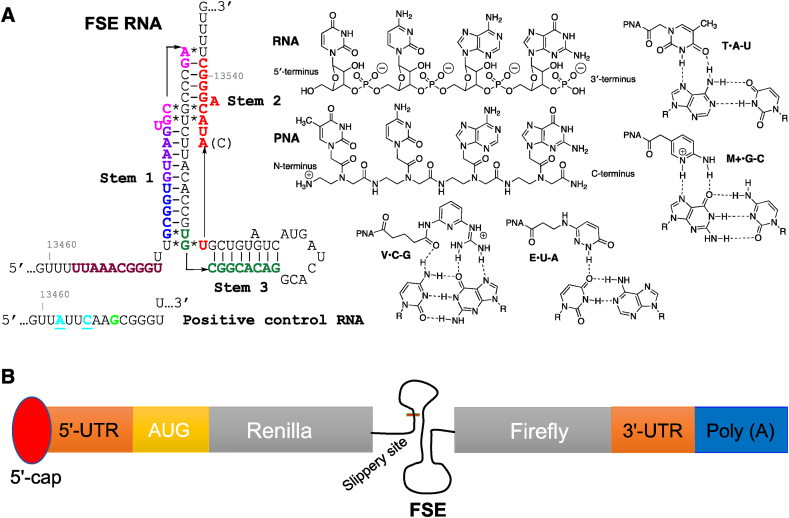
(A) Secondary structure of SARS-CoV-2 FSE with PNA tail-clamp target sequences color coded and structures of PNA and Hoogsteen triplets formed by native and modified nucleobases used in this study. (B) Graphical representation of dual luciferase assay.

The frameshifting stimulatory element (FSE) is a common functional RNA motif found in many viruses, including SARS-CoV-2 (Fig. 1A) and HIV-1 [4,[7], [8], [9]]. A folded RNA structure in the FSE pauses the ribosome at a polyU region upstream of the folded structure called the slippery site. Pausing can induce frameshifting and altered protein synthesis with a frequency that is dependent on the sequence and structure of the folded RNA. The frameshifting frequency is critical for balancing the amounts of different types of viral proteins produced in host cells. In SARS-CoV-2, programmed −1 ribosomal frameshifting occurs in the first open reading frame and is controlled by a *cis*-acting pseudoknot (Fig. 1A). The first reading frame encodes for 16 non-structural proteins with coding regions divided based on their position before (ORF1a) or after (ORF1b) the FSE. The FSE shifts the reading frame by one base in the 5′-direction at the interface between ORF1a and ORF1b during translation. If no shift occurs, only ORF1a is translated resulting in the production of polyprotein 1a (pp1a). Conversely, if frameshifting occurs, both the ORF1a and ORF1b regions are translated resulting in the synthesis of polyprotein 1 ab (pp1ab) [4]. The first polyprotein, pp1a, encodes proteases and other proteins associated with suppressing the host immune response [4]. The second polyprotein, pp1ab, is a longer, frame-shifted polyprotein, which contains the proteins from ORF1a as well as multiple RNA-associated enzymes, including the RNA-dependent RNA polymerase [4]. The importance of the FSE is highlighted by the high sequence conservation between various coronaviruses; for example, SARS-CoV-2 and SARS-CoV FSE pseudoknots differ by only one nucleotide. SARS-CoV-2 has adenosine while SARS-CoV has cytosine at position 13533.

Various studies have proposed different secondary and tertiary structures of SARS-CoV-2 FSE RNA. Alternative RNA conformations were found for shorter model sequences when compared to full length viral RNA in cells [5,6]. Early work on the FSE in coronaviruses proposed a three-stemmed, H-type pseudoknot structure based on RNA secondary structure predictions, nuclease mapping, and mutational analysis [4]. Recent cryo-EM structure [10] of the ribosome arrested on mRNA in a pre-frameshifting state and a crystal structure [11] of short FSE RNA complexed with a protein chaperon both revealed a similar secondary structure that was different than the originally proposed FSE [12]. Another cryo-EM structure of a shorter FSE RNA sequence (88 nucleotides long) reported by Zhang et al. [9] was consistent with the originally proposed secondary structure of pseudoknot but suggested an unusual tertiary fold where the 5′ end of RNA is threaded through a ring formed by the three-stem pseudoknot. Collectively, the structural, computational [13], and biophysical [14] studies confirm that the shorter model sequences of FSE fold in a dynamic ensemble of alternative structures.

Recent DMS-MaPseq [15] and SHAPE-MaP [16] studies of the entire SARS-CoV-2 genomic RNA in infected cells revealed new alternative structures of FSE that involve long-range interactions with viral RNA outside of the originally proposed FSE sequence. These studies also demonstrated that the FSE folds into alternative dynamic structures and cannot be accurately described as a single static secondary structure. In one study, the original three-stemmed, H-type pseudoknot was a minor conformer in observed ensembles [16]. Taken together with studies in shorter model sequences, these results illustrate the complexity of the FSE folding landscape in biological systems.

The critical role that FSE plays in biology of SARS-CoV-2 makes it a highly attractive drug target [17]. Small molecule inhibitors of SARS-CoV-2 frameshifting have been identified and proposed as potential leads for development of antiviral therapeutics [8,10]. Innovative approaches based on RNA binding and ribonuclease recruiting small molecules (RIBOTACs) have given promising results [18]. Alternatively, antisense oligonucleotides have also been suggested as promising candidates for therapeutic targeting of FSE RNA [9,19]. Most relevant to the present study, previous work using antisense peptide nucleic acids (PNAs, Fig. 1A) showed that disruption of the FSE structure led to ∼94 % reduction in frameshifting at 5 μM PNA concentration in a dual luciferase assay in Vero cells and inhibited non-infectious SARS-CoV replicons in HEK293 cells with an IC_50_ of 4.4 μM [19]. A more recent study by Zhang et al. [9] demonstrated the ability for antisense locked nucleic acids (LNA) to inhibit SARS-CoV-2 FSE by binding to stems 1, 2, and 3. LNAs binding to stem 1 elicited the strongest inhibition. In the present study, using a cell-free, dual luciferase assay (Fig. 1B) we found that PNAs targeting stem 2 of FSE RNA (color coded red in Fig. 1A) were stronger inhibitors of protein translation and frameshifting than PNAs binding to stem 1, stem 3, or the slippery site. Surprisingly, a duplex-forming antisense PNA was a stronger inhibitor of frameshifting than a PNA tail-clamp with the same sequence, despite the higher thermal stability of the PNA-RNA-PNA triplexes formed by the latter. Unexpectedly, the addition of cationic triplex-forming PNAs to duplex-forming antisense PNAs caused a strong and sequence non-specific enhancement of inhibition of translation and frameshifting.

## 2 Material and methods

### 2.1 Peptide nucleic acid (PNA) synthesis

PNA synthesis was performed on an Expedite 8909 DNA/RNA/PNA synthesizer at 2 μmol scale on NovaSyn® TG Sieber resin (Novabiochem), using methods previously developed by our group [20,21]. PNA tail-clamps were synthesized on TentaGel® XV RAM (Rapp Polymere) resin to improve the purity of crude PNAs and purified PNA yields. For synthesis using TentaGel® XV RAM resin, the same coupling procedures were used as with the NovaSyn® TG Sieber resin, except for the first coupling. The first coupling was performed as a double coupling with increased coupling times from 5 min to 10 min. Commercial A, G, T, C, and AEEA PNA monomers were purchased from Link Technologies or from PolyOrg. Synthesis of PNAs containing the modified monomers M [20,21], V [22], and E [23,24] has been previously described by our group.

Monomers solutions for PNA synthesis were made in oven-dried amber bottles in dry *N*-methyl-2-pyrrolidone (NMP) at a concentration of 0.2 M. All PNA synthesis reagents (deblock, cap, base, and activator) were made in oven-dried amber bottles with dry DMF. Deblock was a solution of 20 % (v/v) piperidine in DMF. Cap was a mixture of 6 % (v/v) lutidine and 5 % (v/v) acetic anhydride in DMF. HBTU activator was dissolved in DMF to a concentration of 0.18 M. Base was a mixture of 3 % (v/v) lutidine (0.27 M) and 2 % (v/v) *N,N*-diisopropylethylamine (0.12 M) in DMF. All solutions were adequately purged with N_2_ before solid-phase PNA synthesis. After synthesis, PNAs were cleaved from solid support using 0.6 mL of cleavage solution (95:5:2:2 mixture of TFA/phenol/triisopropylsilane/water) for 2 h using two-syringe pull-push method. PNA cleavage solution was subsequently separated into three 1.5 mL Eppendorf tubes (200 μL in each), the resin was washed once more with an additional 0.2 mL of the cleavage solution. PNAs were precipitated by the addition of chilled diethyl ether (∼1.0 mL) and incubation at −20 °C for 15 min followed by the centrifugation (15000 rpm, 30 min). Diethyl ether was then removed using a pipette and the PNA was suspended once more in 1.0 mL diethyl ether and centrifuged (15000 rpm, 10 min). The diethyl ether was again decanted and the crude PNAs, white pellets, were dissolved in 1.25 mL of 0.1 % formic acid in water. Samples were then analyzed by LC/MS to confirm the correct sequence, purified by reverse-phase HPLC, and finally reanalyzed by LC/MS to confirm their purity. The conditions of HPLC and LC/MS experiments are given in Figs. S1–S21 in Supporting Information. Yields of purified PNAs were quantified by measuring the absorbance at 260 nm and using extinction coefficients of 15,400 M^−1^cm^−1^ for A, 11,500 M^−1^cm^−1^ for G, 8560 M^−1^cm^−1^ for T, 7400 M^−1^cm^−1^ for C, 806 M^−1^cm^−1^ for M, 4200 M^−1^cm^−1^ for V, and 5984 M^−1^cm^−1^ for E. After PNA quantification, the solutions were lyophilized and redissolved in nuclease-free water to a final concentration of 240 μM.

### 2.2 Dual luciferase assay

In the dual luciferase assay, the SARS-CoV-2 FSE is inserted in mRNA between the Rluc (Renilla) and Fluc (Firefly) luciferase sequences. The gene encoding Rluc is placed in the 0 frame upstream of the FSE, whereas the Fluc gene is placed in the −1 frame downstream of the FSE (Fig. 1B). The assay was performed following the procedures reported by Zhang et al. [9]. The frameshifting efficiency was measured by comparing the Rluc and Fluc signals in the presence and absence of PNA ligands (Fig. 1A). We also constructed a positive control RNA (PC RNA), as reported by Zhang et al. [9], where a single-nucleotide insertion (green G in Fig. 1A) and two nucleotide mutations (U and A to turquoise A and C, respectively) disrupted the slippery sequence and placed Fluc in the same reading frame as Rluc.

**Plasmid construction and mRNA synthesis:** Bicistronic plasmid (p2luc) was purchased from NovoPro Bioscience, Shanghai, China. The SARS-CoV-2 frameshifting element (FSE) was inserted into the vector via Sa1I and SacI restriction sites. The FSE was positioned between the Rluc and Fluc luciferase sequences, with Fluc positioned in the −1 frame compared to Rluc. A positive control plasmid was synthesized with a single-nucleotide insertion (green G in Fig. 1A) in addition to a disrupted slippery sequence (mutation of U and A to turquoise A and C, respectively) that caused Fluc to be in the zero-frame relative to Rluc [9]. The correct sequences of all plasmids were confirmed by Sanger sequencing. Plasmid DNA was linearized using the HindIII-HF enzyme and purified using phenol-chloroform extraction for *in vitro* transcription. mRNA was synthesized at 37 °C for 1 h using the mMESSAGE mMACHINE T7 transcription kit (Invitrogen) according to the manufacturer's recommendations. Template DNA was removed using DNase I (Thermo Scientific) and purified using the RNeasy MinElute Cleanup Kit (QIAGEN). Finally, the concentration of mRNA was measured using Nanodrop (Thermo Scientific).

***In vitro* frameshifting assay:** 500 ng mRNAs were mixed with 5.5, 5.2, 4.2, 3.0, 4.7, 4.0, 2.5, 4.2, 4.8, 4.2, 3.6, 3.0, and 1.7 μL nuclease-free water and 1.5 μL of 5 × folding buffer containing 30 mM HEPES (pH 6.95), 100 mM KCl, and 0.01 % CHAPS. The mixture was heated at 85 °C for 5 min and further incubated at 37 °C for 5 min. To this mixture, 0.0, 0.3, 1.3, 2.5, 0.8, 1.5, 3.0, 1.3, 0.7, 1.3, 1.9, 2.5, and 3.8 μL of PNA were added to reach various final concentrations (0, 1, 5, 10, 25, 50, 100, 250, 500, 750, 1000, and 1500 nM). The mixture was incubated at 37 °C for 30 min to enable folding. Rabbit Reticulocyte lysate system was prepared by mixing 5.6 μL cell lysate, 0.13 μL amino acid mixture minus methionine, 0.13 μL amino acid mixture minus leucine, and 0.13 μL RNaseOut. Then, 2 μL of the folded RNA solution was mixed with 6 μL of Rabbit Reticulocyte lysate system and the mixture was incubated at 30 °C for 1.5 h. The translation products were stored at 4 °C until luminescence was measured.

The luciferase assay was performed using the Dual-Glo® Luciferase Assay System according to the manufacturer's recommendations (Fig. S22). Briefly, in a 96-well plate, 2 μL of translation product was mixed with 18 μL of 1 × passive lysis buffer. To this mixture, 20 μL of luciferase assay reagent was added, and the mixture was incubated at room temperature for 10 min with constant shaking on a 3D shaker. Firefly luminescence was measured with the GLOMAX microplate luminometer (Promega). Next, 20 μL of Stop & Glo Reagent (a 1:100 mixture of Stop & Glo substrate and Stop & Glo buffer) was added to each sample well. This solution was further incubated for 10 min, and Renilla luminescence was measured.

The inhibition of Fluc gene translation was calculated as follows:

$$\text{Fluc}\;\text{gene}\;\text{translation}=\frac{\text{FlucS}-\text{Blank}}{\text{RlucS}-\text{Blank}}\times 100\%$$

The percent of frameshifting was calculated as follows:

$$\text{Frameshifting}=\frac{\frac{\left(\text{FlucS}-\text{Blank}\right)}{\left(\text{RlucS}-\text{Blank}\right)}}{\frac{\left(\text{FlucC}-\text{Blank}\right)}{\left(\text{RlucC}-\text{Blank}\right)}}\times 100\%$$

FlucS and RlucS are the Firefly and Renilla luminescence of mRNA with the wildtype frameshifting element, whereas FlucC and RlucC are the Firefly and Renilla luminescence of positive control mRNA with a disrupted slippery site. The IC_50_ was calculated using GraphPad Prism 9 (Figs. S24–S27).

### 2.3 UV-melting experiments

UV-melting experiments were performed on a Shimadzu UV-2600 spectrophotometer equipped with a TMSPC-8 temperature controller. A temperature ramp rate of 1.0 °C per minute was used and the absorbance was monitored at 260 nm (Fig. S28). Experiments were performed on 2.5 μM PNAs and 2.5 μM single-stranded RNA target (ssRNA_2) in a buffer containing 50 mM potassium phosphate, 2 mM MgCl_2_, 90 mM KCl, 10 mM NaCl at pH 7.4. Solutions were prepared as follows. Stock solutions of both PNA and ssRNA_2 (2.3 μL of 240 μM each) were combined, frozen, and lyophilized. Buffer (230 μL) was added to the pellet, followed by vortexing and centrifugation. The solution was then heated to 95 °C for 5 min and then snap-cooled to 4 °C and left for 5 min. Samples were again vortexed and centrifuged, and then distributed to two cells (115 μL in each) of the eight-cell cuvette (path length 10 mm). Samples were then heated three times from 10 °C to 95 °C. The melting temperatures (*T*_m_) were obtained using Shimadzu Lab Solutions *T*_m_ Analysis software. Three of the replicates were used to determine average and standard deviation in *T*_m_ (Table S2).

### 2.4 Isothermal titration calorimetry

Isothermal titration calorimetry experiments were performed on a MicroCal ITC200 instrument at 25 °C in 50 mM potassium phosphate buffer (pH 7.4) containing 2 mM MgCl_2_, 90 mM KCl, 10 mM NaCl. Solutions were prepared as follows. Stock solutions of PNA (20 μL of 240 μM) and ssRNA_2 (12.5 μL of 240 μM) were aliquoted and lyophilized. 64 μL of buffer was added to the PNA pellet and 300 μL of buffer was added to the RNA pellet. Solutions were then vortexed and centrifuged to allow for proper mixing. The RNA solution was then heated to 95 °C for 5 min and then snap-cooled to 4 °C and left for 5 min to allow for proper folding. Standard concentration of PNA was 75 μM and stand concentration of RNA was 10 μM. Aliquots of PNA solution (2.45 μL) were sequentially injected from a 40 μL rotating syringe (750 rmp) into 200 μL of RNA solution (Figs. S29–S32). Results were analyzed using MicroCal PEAQ-ITC software (Table S3).

## 3 Results

In the present study, we explored how the disruption of the pseudoknot structure using PNA tail-clamps affects frameshifting of SARS-CoV-2 mRNA. We hypothesized that binding of PNA tail-clamps would rearrange the double helical structure of the FSE pseudoknot necessary to induce the frameshifting, resulting in inhibition of frameshifting. Our hypothesis was supported by results of previous studies using antisense PNA [19] and LNA [9] ligands. To study frameshifting frequency of SARS-CoV-2 RNA, we used the cell-free *in vitro* dual luciferase frameshifting assay that was previously reported by Zhang et al. [9].

**Fig. 2:**
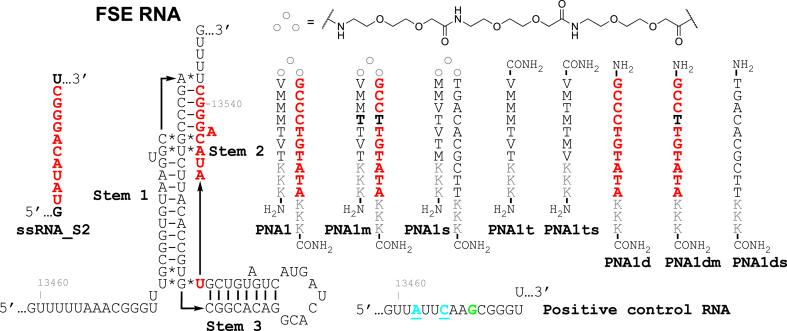
Secondary structure of SARS-CoV-2 FSE with the PNA1 tail-clamp target sequence highlighted in red. Sequences of tail-clamp PNA1 targeting Stem 2 and control PNAs.

To study the effect of PNA tail-clamps on frameshifting of SARS-CoV-2 RNA, we selected five purine rich sequences spanning nucleotides 13459 to 13546 of FSE RNA (color coded in Fig. 1A) that were considered good targets for PNA tail-clamps. Each target sequence was 10 nucleotides long, had six nucleotides long Hoogsteen triplex region, and had a four nucleotides long Watson-Crick duplex tail region (Fig. 2, Fig. 4). The Hoogsteen triplexes were formed using our previously developed triplex-forming PNAs [[25], [26], [27]]. Binding of the triplex strand is driven predominantly by thymine (T) forming canonical T•A-U triplets and 2-aminopyridine (M, Fig. 1A) forming exceptionally stable M+•G-C triplets at physiological conditions. Each Hoogsteen region also contained two pyrimidine interruptions that were recognized using previously developed modified nucleobases V [22] and E [23,24] that formed the corresponding V•C-G and E•U-A triplets (Fig. 1A).

**Table 1:**
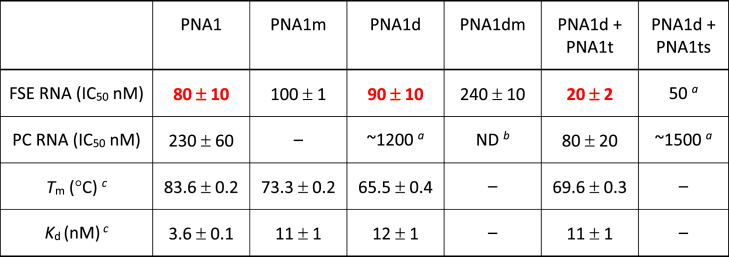
Results of the dual luciferase assay and binding affinity of PNA1 and control PNAs.

### 3.1 PNAs targeting stem 2 of SARS-CoV-2 FSE RNA inhibit frameshifting

We started our study with PNAs targeting stem 2 of SARS-CoV-2 FSE RNA (red in Fig. 1, Fig. 2). The frameshifting frequency of FSE RNA in the presence and absence of PNA ligands was measured using the cell-free, *in vitro*, dual luciferase frameshifting assay (Table 1). The binding affinity of PNAs was measured using UV thermal melting (Table 1, *T*_m_, °C) and isothermal titration calorimetry (Table 1, *K*_d_, nM) of PNA complexes with single-stranded RNA target (ssRNA_S2). The binding site of PNA1 (nucleotides 13532–13541) partially overlapped with the binding site of antisense PNA used by Oh and co-workers (nucleotides 13528–13542) [19].

Binding of tail-clamp PNA1 to the FSE RNA inhibited the Fluc gene translation with IC_50_ 80 nM (Table 1) as measured by the ratio of Renilla and Firefly luciferase signals. The inhibition was at least in part due to disruption of the FSE structure because binding of PNA1 to the positive control RNA (PC RNA) resulted in ∼3 fold higher IC_50_. In the PC RNA sequence (Fig. 2) both Renilla and Firefly luciferases were in the same reading frame. Therefore, the effect of PNAs on PC RNA was due to direct inhibition of translating ribosome, most likely due to a steric block by the PNA-RNA-PNA triplex. Introduction of a mutation in the clamp sequence (bold Ts in PNA1m) resulted in a minor change of IC_50_, which was not surprising because it disrupts only the triplex part while creating a wobble T-G base pair on the Watson-Crick strand. Interestingly, the duplex-forming PNA1d had a similar IC_50_ (90 nM) when compared to the full clamp, and the activity was notably reduced by a C to T mutation in PNA1dm. The inhibition by PNA1d was largely due to disruption of the FSE structure because binding of PNA1d to positive control RNA (PC RNA) had a weak effect on translation with an IC_50_ of ∼1200 nM. The inhibition was sequence selective and driven primarily by the Watson-Crick PNA-RNA duplex because neither the scrambled clamp (PNA1s, Fig. 2) nor the triplex-forming PNA alone (PNA1t) showed any activity. Surprisingly, combining the duplex- and triplex-forming PNAs together (PNA1d + PNA1t) gave the strongest inhibition with an IC_50_ of 20 nM. Control experiments that combined duplex-forming PNA1d with a scrambled sequence of triplex-forming PNA1ts gave a slightly reduced IC_50_ of ∼50 nM, which strongly suggested that the unusual inhibition by the combination of two individual PNAs was caused by some additional sequence non-specific interactions.

Single-stranded ssRNA_S2 (Fig. 2) models the target site of PNA1 in stem 2 of the FSE. Measurements of binding affinities of PNAs to ssRNA_S2 showed no clear correlation between thermodynamic stability of PNA-RNA complexes and frameshifting inhibition (Table 1). As expected, the matched tail-clamp PNA1 formed the most stable complex with ssRNA_S2 (highest *T*_m_ and lowest *K*_d_). The binding affinity decreased notably for the mismatched PNA1m. The duplex only control PNA1d had the lowest affinity despite having a comparable IC_50_ with PNA1. The most potent inhibitor, the combination of PNA1d + PNA1t had an intermediate *T*_m_ and *K*_d_ when complexed with ssRNA_S2. Overall, the binding affinity measurements did not provide insights into the unexpected frameshifting inhibition afforded by the combination of PNA1d + PNA1t.

**Fig. 3:**
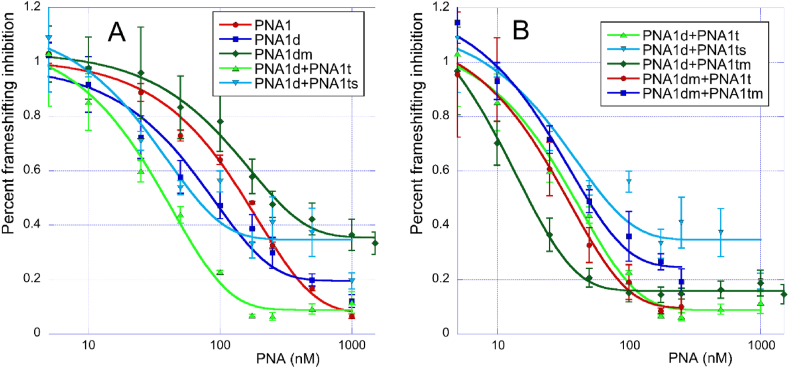
Normalized inhibition of frameshifting by various PNAs targeting stem 2 of SARS-CoV-2 FSE. (A) Comparison of tail-clamp PNA1 with various control PNAs. (B) The sequence non-specific effect of combining various triplex-forming PNAs with PNA1d and PNA1dm.

In the previous study, Zhang et al. [9] used the positive control RNA (PC RNA) to normalize the results of dual luciferase assay and separate the frameshifting inhibition from direct inhibition of protein synthesis due to steric block by LNA antisense oligonucleotide-RNA complexes. When we applied the normalization to our data on PNAs, the results (Fig. 3) showed similar trends as in Table 1. The combination of PNA1d + PNA1t was the strongest inhibitor of frameshifting (light green data in Fig. 3A). The inhibition was slightly reduced by using the scrambled triplex-forming PNA in PNA1d + PNA1ts (light blue data in Fig. 3A).

**Fig. 4:**
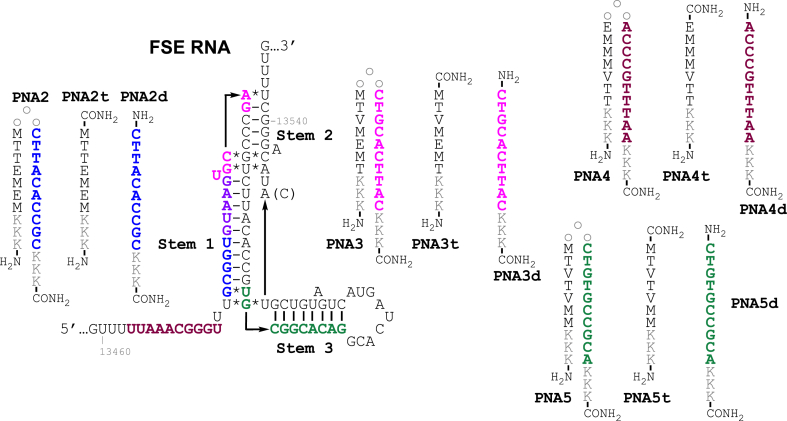
Secondary structure of SARS-CoV-2 FSE with PNA tail-clamp target sequences color coded. Sequences of tail-clamps PNA2-PNA5 and control PNAs.

**Table 2:**
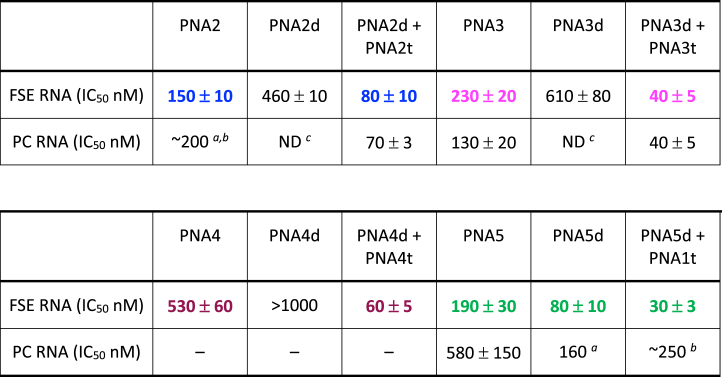
Results of the dual luciferase assay of PNA2-PNA5 and control PNAs.

Interestingly, the duplex-forming PNA1d appeared to be a stronger inhibitor of frameshifting than the tail-clamp PNA1 (c.f. dark blue and red data in Fig. 3A). This result is surprising because PNA1 had a notably higher binding affinity to single-stranded RNA (Table 1). The frameshifting inhibition by the duplex-forming PNA1d was highly sequence specific because introduction of the most stable mismatch, T-G wobble pair, instead of the fully matched C-G in PNA1dm, strongly reduced the inhibition (c.f. dark blue and dark green data in Fig. 3A). The fully matched PNA/RNA complexes generally achieved more complete inhibition (I_max_, 80–95 % in Fig. 3A) while complexes containing a mismatch resulted in a less complete inhibition (65–80 %) at high nM to low μM concentrations. Among the combinations of two PNAs, PNA1d + PNA1tm and PNA1dm + PNA1t deviate from this trend, giving I_max_ values of >80 % and ∼90 % (Fig. 3B), respectively. The lack of sequence specificity when adding a separate triplex-forming PNA to PNA1d was further confirmed by comparing various combinations of the two PNAs (Fig. 3B). The strongest frameshifting inhibition was observed when the matched PNA1d was combined with the mismatched PNA1tm (dark green in Fig. 3B). All other combinations showed similar inhibition activities irrespective of whether the mismatched bases were in PNA1d or PNA1t sequences.

### 3.2 PNAs targeting the slippery site, stem 1, and stem 3

A previous study [9] using the *in vitro* frameshifting assay showed that LNA antisense oligonucleotides targeting stem 1 (Fig. 4) had greater inhibitory effect than LNAs targeting stems 2 and 3. To explore the effect of PNA binding to other parts of FSE, we designed two PNA clamps targeting stem 1 and two PNA clamps targeting the slippery sequence and stem 3, respectively (Fig. 4). Binding of PNA2 and PNA3 to stem 1 of the FSE caused a notably weaker inhibition of translation than the binding of PNA1 to stem 2 (c.f., Table 1, Table 2). For both PNA2 and PNA3, the IC_50_ for inhibition was like the IC_50_ of steric blocking of ribosome on PC RNA. Duplex-forming PNAs alone had significantly lower activity. Consistent with results when targeting stem 2 (Table 1), combining the duplex- and triplex-forming PNAs together gave the strongest inhibition, with an IC_50_ of 80 nM for PNA2d + PNA2t and an IC_50_ of 40 nM for PNA3d + PNA3t (Table 2). For both combinations, the IC_50_ for inhibition was identical, within the error limits, to the IC_50_ of steric blocking of ribosome on the PC RNA. Binding of PNA4 to the slippery sequence (maroon in Fig. 4) had the weakest effect among the targets tested (Table 2). Consistent with the overall trend, the combination of PNA4d + PNA4t showed an order of magnitude stronger inhibition than PNA4. Finally, binding of PNA5 to stem 3 gave similar frameshifting inhibition compared to binding of PNA2 and PNA3 to stem 1, with the notable exception that the duplex-forming PNA5d was more active than PNA5. Normalization of activity to separate the frameshifting inhibition from direct inhibition of protein synthesis showed that PNA5 and PNA5d had a relatively weak effect on frameshifting of SARS-CoV-2 RNA (Fig. S23) compared to PNA1 and PNA1d targeting stem 2.

## 4 Discussion

The mechanism by which coronaviruses control the frameshifting in ORF1a/b translation is not completely understood. However, the correct timing and ratio of pp1a and pp1ab proteins (translated from ORF1a and ORF1a/1b, respectively) is critical for viral viability and pathogenesis [1,2]. Since translation of pp1a and pp1ab is controlled through the programmed −1 ribosomal frameshifting, the *cis*-acting pseudoknot of the FSE responsible for the frameshifting is a promising drug target [17]. Moreover, the conserved sequence and structure of FSE, as SARS-CoV-2 and SARS-CoV pseudoknots differ by only one nucleotide (A in SARS-CoV-2 instead of C in SARS-CoV at position 13533), is expected to limit the ability of the virus to evolve drug resistant mutants. Several studies have screened known drugs and novel compounds with the goal of identifying promising antiviral inhibitors of SARS-CoV-2 [17]. Small molecule inhibitors of SARS-CoV-2 frameshifting have been identified and proposed as potential leads for development of antiviral therapeutics [8,10].

Alternatively, antisense oligonucleotides have been suggested as promising candidates for therapeutic targeting of the FSE RNA [9,19]. Most relevant to the present study, previous work by Oh and co-workers using an antisense PNA targeting the stem 2/loop 3/stem 3 region of SARS-CoV FSE showed that disruption of the pseudoknot structure led to ∼94 % reduction in frameshifting at 5 μM PNA concentration in a dual luciferase assay in Vero cells [19]. In the same study, the antisense PNA inhibited non-infectious SARS-CoV replicons in HEK293 cells with an IC_50_ of 4.4 μM [19]. In contrast, a recent study by Zhang et al. [9] found that targeting stem 1 with antisense LNA inhibited frameshifting (in a cell-free dual luciferase assay) and SARS-CoV-2 replication (in ACE2-A549 cell line) to a greater extent than targeting stems 2 or 3.

In the present study, we found that targeting SARS-CoV-2 FSE with PNA tail-clamps gave several unexpected results. The first surprising result was that the PNA tail-clam PNA1 and the antisense part (duplex only) PNA1d showed similar activity (Table 1). After normalization using the positive control (PC RNA), antisense PNA1d appeared to be a stronger inhibitor of frameshifting than tail-clamp PNA1 (c.f., blue and red lines in Fig. 3). However, PNA1 reached more complete inhibition (I_max_ value of >90 % in Fig. 3A) than PNA1d (I_max_ value of ∼80 % in Fig. 3A). This result was unexpected because PNA1 had higher thermodynamic affinity for the single-stranded RNA target than PNA1d, as demonstrated by the 18 °C higher *T*_m_ and three-fold lower *K*_d_ (Table 1). Interestingly, a similar result was observed for PNA5 and PNA5d (Table 2) targeting stem 3 (Fig. 4). In the case of PNA2 and PNA3 targeting stem 1 and stem 1-2 junction, respectively, the tail-clamps were more active than the antisense strands. However, translation inhibitions afforded by PNA2 and PNA3 was caused by direct steric interference with the ribosome and not frameshifting inhibition because both PNAs had similar effect on PC RNA. It should be noted that the thermodynamic stability of PNA-RNA complexes was measured on a short, single-stranded RNA target and, therefore, did not account for binding to the folded FSE RNA target. It is conceivable that the lower activity of PNA1 (as well as PNA2, PNA3, and PNA5) was caused by higher barriers for invasion of FSE secondary structure for the larger PNA tail-clamps than for the smaller antisense PNAs. Consistent with this notion, PNA4 that targets single-stranded slippery site had a higher activity than PNA4d.

Another unexpected result was that the combination of individual antisense and triplex strands PNA1d and PNA1t had the highest activity (Table 1) and was a stronger inhibitor of frameshifting than either PNA1 or PNA1d individually (Fig. 3A). This unusual result was caused by sequence non-specific interactions because the combination of PNA1d with mismatched PNA1tm was the strongest inhibitor of frameshifting among all PNAs tested (dark green line in Fig. 3B). Other combinations of mismatched PNAs, including PNA1d with scrambled PNA1ts also showed unusually strong inhibition of frameshifting (Fig. 3B and Fig. S23). It is conceivable that these non-specific interactions are caused by the highly cationic nature of PNA1t and its variants having two guanidinium groups (part of V nucleobases), three lysines, and three partially protonated M nucleobases. In our previous study [22], we reported that a short triplex-forming PNA containing three V bases in addition to M bases bound strongly and indiscriminately to double-stranded RNAs. Therefore, it is possible that the cationic PNAs were interacting with off-target sequences in SARS-CoV-2 FSE and causing the unexpected “stimulatory” effect on frameshifting inhibition. Consistent with this notion, PNA2t lacking the guanidinium groups had the smallest “stimulatory” effect among the various triplex-forming PNAs (c.f., PNA2 and PNA2 + PNA2t in Table 2).

While PNA clamps have been extensively studied as ligands for single- and double-stranded DNA [28], their binding to RNA is less understood. Early studies by Knudsen and Nielsen [29] showed that pyrimidine rich PNAs and PNA clamps forming PNA_2_-RNA triplexes in the coding region of mRNA inhibited translation, while PNAs forming PNA-RNA duplexes were not able to block the translating ribosome. Another earlier study reported that binding of symmetrical PNA clamps (having the same length of Watson-Crick and Hoogsteen parts) to single-stranded RNA inhibited HIV reverse transcriptase [30]. In contrast to our results, these studies showed that PNA clamps were more effective than combined Watson-Crick and Hoogsteen PNAs. A more recent study showed that PNAs as short as 10 to 13-mers invaded structured regions of Ha-*ras* mRNA and arrested translation by forming highly stable 2:1 PNA-RNA complexes [31]. The complexes were hypothesized to have both Watson-Crick and Hoogsteen base pairings, similar to the PNA tail-clamps used in our study. Most recently, Strömberg and co-workers described invasion of an RNA hairpin that models miR-376b precursor by PNA tail-clamps [32]. Collectively, these previous studies suggested that PNA tail-clamps were more effective in binding and modulating of biological functions of targeted RNAs. However, systematic biophysical studies on PNA tail-clamp invasion and binding to complex double-stranded RNA are lacking. Our surprising results that an antisense PNA was a greater inhibitor of SARS-CoV-2 frameshifting emphasize both the need for more fundamental studies in simple model systems as well as complexities of binding highly structured and tightly folded RNA targets, such as viral pseudoknots.

## 5 Conclusions

In the present study, we explored inhibition of programmed −1 ribosomal frameshifting using PNAs binding to five distinct target sequences in SARS-CoV-2 FSE RNA. The key findings of our study were that: 1) PNAs binding to stem 2 of the FSE RNA inhibited protein translation and frameshifting more effectively than PNAs binding to stem 1, stem 3, or the slippery site; 2) antisense PNAs were stronger inhibitors than PNA tail-clamps, despite higher thermal stability of the PNA-RNA-PNA triplexes formed by the PNA tail-clamps; and 3) combination of cationic triplex-forming PNAs with duplex-forming antisense PNAs caused strong and sequence non-specific enhancement of inhibition of translation and frameshifting.

Our results showed that binding of tail-clamp PNA1 and antisense PNA1d to stem 2 of FSE caused the strongest sequence specific inhibition of protein translation in the cell-free dual luciferase assay. Normalization of the data against inhibition of positive control RNA that separated frameshifting inhibition from direct steric interference with ribosome translation showed that PNA1d was a stronger sequence specific inhibitor of frameshifting than PNA1. This result was counterintuitive because PNA1 had higher thermodynamic affinity for single-stranded RNA than PNA1d. This result underscores the challenges of binding antisense ligands to highly structured and tightly folded RNA targets, such as SARS-CoV-2 pseudoknots. Surprisingly, addition of triplex-forming PNA strands to antisense PNA caused strong sequence non-specific inhibition of frameshifting. This result emphasizes the intricacy of interactions that highly cationic ligands have with RNA targets in complex biological systems.

Our study was limited due to a relatively short RNA model system in a cell-free dual luciferase assay. In our study, we designed the FSE RNA model sequence and PNA ligands based on the cryo-EM structure reported by Zhang et al. [9]. We used 88 nucleotides long fragment (nucleotides 13459 to 13546) of SARS-CoV-2 FSE RNA inserted between the Renilla and Firefly luciferase sequences in a 2690 nucleotides long mRNA. As discussed in Introduction, various studies have found different FSE structures depending on the length of RNA studied. Moreover, structural studies of the entire SARS-CoV-2 genomic RNA in infected cells have found alternative structures of the FSE that involve long-range interactions with viral RNA outside of the originally proposed shorter FSE sequence.

Overall, our results on targeting SARS-CoV-2 FSE RNA revealed significant gaps in our knowledge on targeting RNA using PNA tail-clamps and underscore the need for further fundamental studies on RNA recognition by triplex-forming and tail-clamp PNAs. To address these challenges, the future studies will focus on biophysical characterization of PNA binding to less complex RNA model systems, before designing a new generation of triplex-forming and tail-clamp PNAs as ligands for SARS-CoV-2 FSE. Given the limitations discussed above, it will be advantageous to use a more biologically relevant, non-infectious SARS-CoV-2 replicon in live cells to test the frameshifting inhibition by PNA ligands.

## Data availability statement

The data generated in this study are available in the article and Supporting Information.

## CRediT authorship contribution statement

**Md Motiar Rahman:** Writing – review & editing, Methodology, Investigation, Formal analysis, Data curation. **Christopher A. Ryan:** Writing – review & editing, Methodology, Investigation, Conceptualization. **Brandon R. Tessier:** Writing – review & editing, Investigation. **Eriks Rozners:** Writing – review & editing, Writing – original draft, Validation, Supervision, Resources, Project administration, Funding acquisition, Formal analysis, Data curation, Conceptualization.

## Declaration of competing interest

The authors declare that they have no known competing financial interests or personal relationships that could have appeared to influence the work reported in this paper.
