# Supplementary material for: Peptide nucleic acids (PNAs) control function of SARS-CoV-2 frameshifting stimulatory element trough PNA-RNA-PNA triplex formation
Source: Heliyon. 2024 Jun 29;10(13):e33914. doi: 10.1016/j.heliyon.2024.e33914 (PMC11282987; doi:10.1016/j.heliyon.2024.e33914)
Supplement: Multimedia component 1 [file mmc1.pdf]

Supporting Information

Peptide Nucleic Acids (PNAs) Control Function of SARS-CoV-2 Frameshifting Stimulatory Element Trough PNA-RNA-PNA Triplex Formation

Md Motiar Rahman,‡ Christopher A. Ryan,‡ Brandon R. Tessier, and Eriks Rozners*
Department of Chemistry, Binghamton University, The State University of New York, Binghamton, New York 13902, United States.

Table S1. Molecular weight and de-convoluted masses of PNAs

PNA	$Mass_{(calc)}$	M/Z calc	M/Z found
PNA1	5838.0	730.8, 835.0, 974.0, 1168.6, 1460.5	730.6, 834.8, 973.8, 1168.4, 1460.4
PNA1m	5885.0	736.6, 841.7, 981.8, 1178.0, 1472.3	736.5, 841.6, 981.7, 1177.8, 1472.2
PNA1s	5838.0	730.8, 835.0, 974.0, 1168.6, 1460.5	730.6, 834.9, 973.9, 1168.5, 1460.4
PNA1t	2332.5	334.2, 389.8, 467.5, 584.1, 778.5	334.0, 389.6, 467.3, 583.8, 778.1
PNA1ts	2330.9	389.5, 467.2, 583.7, 777.9, 1166.5	389.5, 467.3, 583.8, 778.1, 1166.6
PNA1d	3088.1	442.2, 515.7, 618.6, 773.0, 1030.4	442.0, 515.5, 618.4, 772.8, 1030.0
PNA1dm	3103.1	518.2, 621.6, 776.8, 1035.4, 1552.6	518.0, 621.4, 776.5, 1034.9, 1552.1
PNA1ds	3088.1	515.7, 618.6, 773.0, 1030.4, 1545.1	515.5, 618.4, 772.8, 1030.1, 1544.6
PNA1tm	2364.5	395.1, 473.9, 592.1, 789.2, 1183.3	394.8, 473.6, 591.8, 788.8, 1182.6
PNA2	5618.4	703.3, 803.6, 937.4, 1124.7, 1405.6	703.2, 803.5, 937.3, 1124.6, 1405.6
PNA2t	2167.9	434.6, 543.0, 723.6, 1085.0	434.4, 542.8, 723.4, 1084.5
PNA2d	3033.0	506.5, 607.6, 759.3, 1012.0, 1517.5	506.3, 607.5, 759.1, 1011.7, 1517.8
PNA3	5711.1	635.6, 714.9, 816.9, 952.9, 1143.2	636.0, 715.4, 817.5, 953.5, 1144.0
PNA3t	2248.5	375.8, 450.7, 563.1, 750.5, 1125.3	375.8, 450.8, 563.3, 750.8, 1125.6
PNA3d	3045.9	508.7, 610.2, 762.5, 1016.3, 1523.9	508.8, 610.4, 762.8, 1016.6, 1524.5
PNA4	5735.1	638.2, 717.9, 820.3, 956.9, 1148.0	638.8, 718.5, 821.0, 957.6, 1149.0
PNA4t	2250.2	451.0, 563.6, 751.1, 1126.1	450.8, 563.3, 750.7, 1125.5
PNA4d	3072.1	513.0, 615.4, 769.0, 1025.0, 1537.0	512.8, 615.2, 768.7, 1024.6, 1536.5
PNA5	5834.5	649.3, 730.3, 834.5, 973.4, 1167.9	649.7, 730.7, 835.0, 973.9, 1168.6
PNA5t	2332.5	389.8, 467.5, 584.1, 778.5, 1167.3	389.5, 467.2, 583.8, 778.2, 1166.6
PNA5d	3089.1	515.8, 618.8, 773.3, 1030.7	515.6, 618.6, 773.0, 1030.2

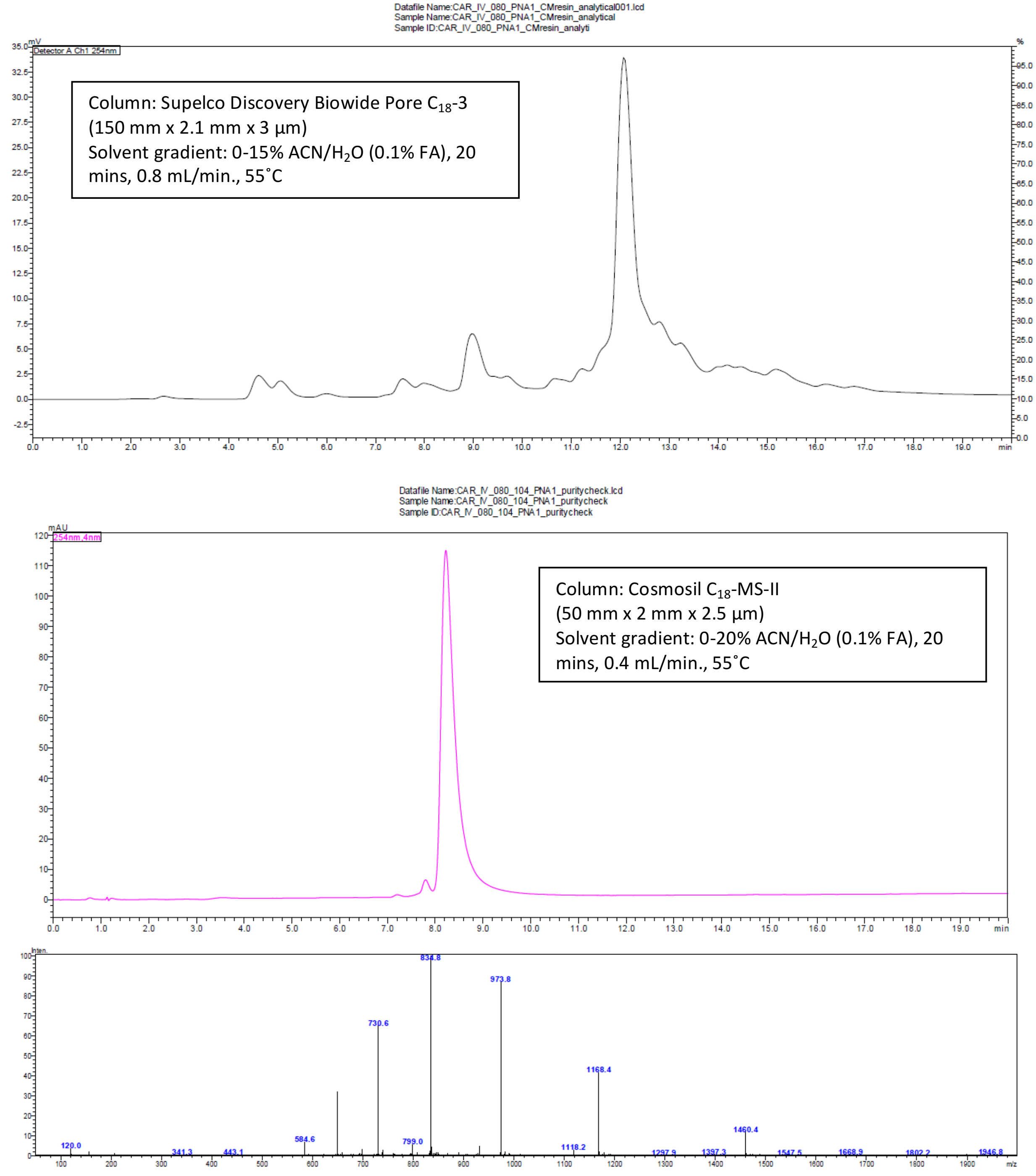

Figure S1. Crude HPLC (top) and purified LC/MS (middle/bottom) data of PNA1.

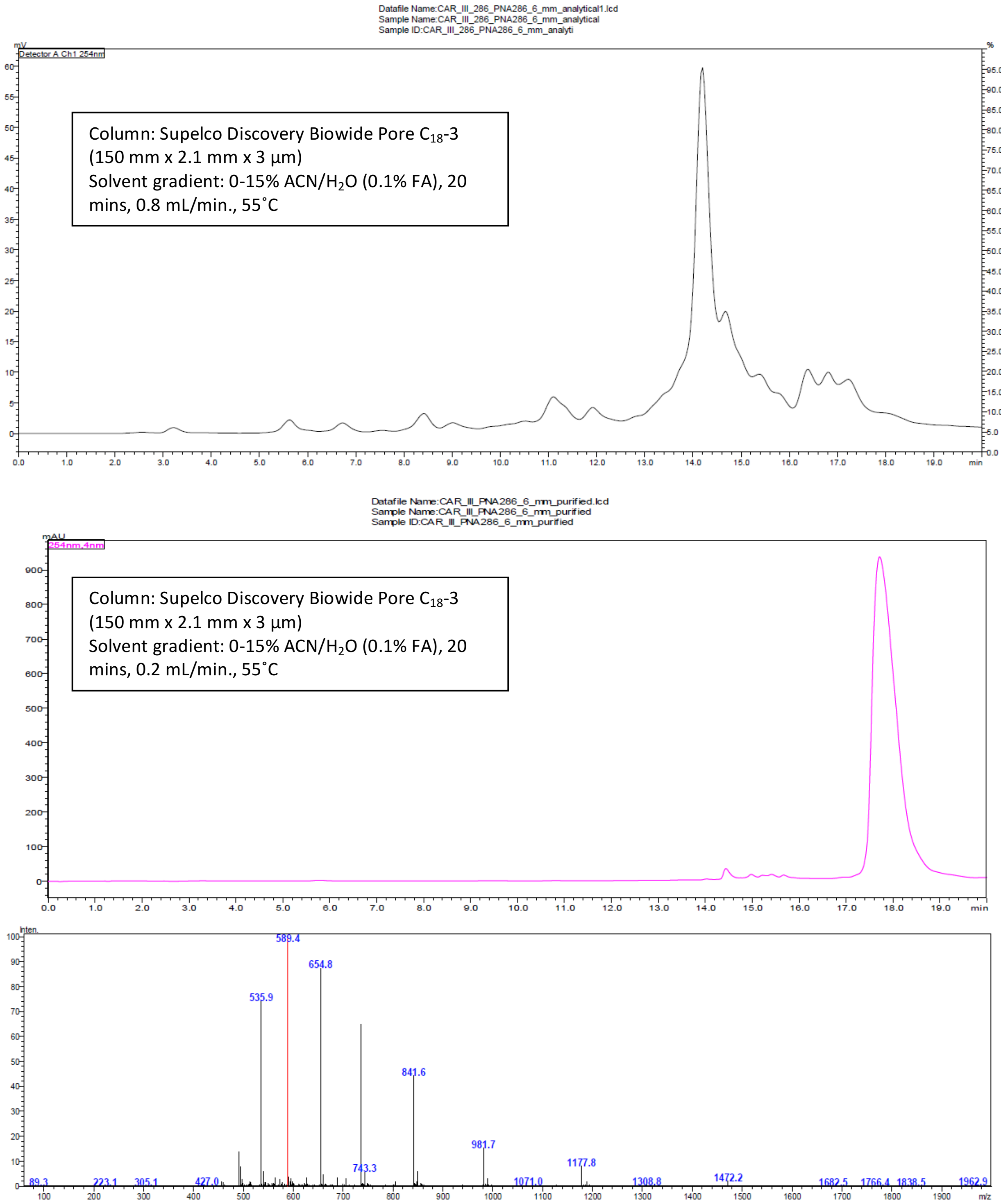

Figure S2. Crude HPLC (top) and purified LC/MS (middle/bottom) data of PNA1m.

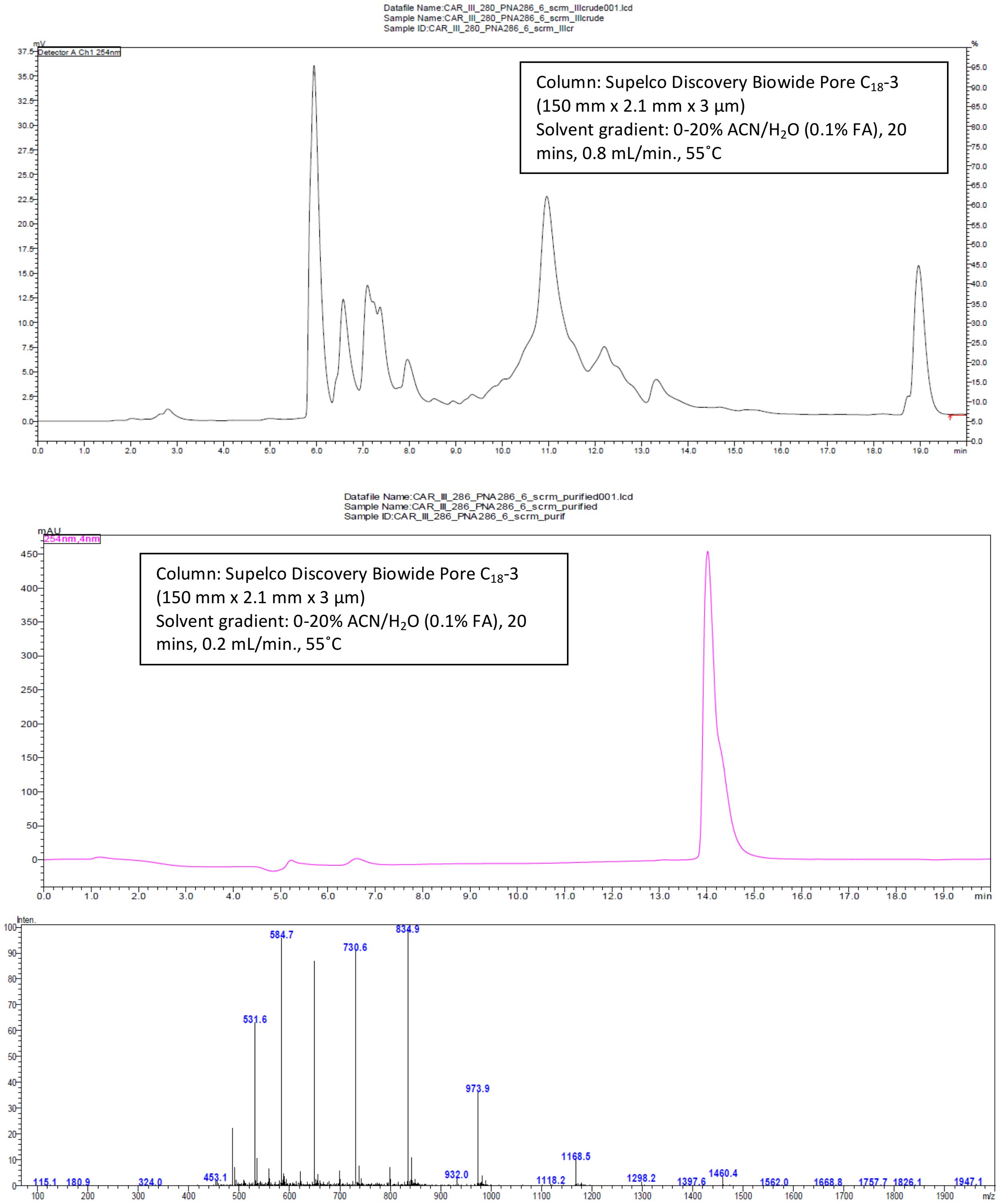

Figure S3. Crude HPLC (top) and purified LC/MS (middle/bottom) data of PNA1s.

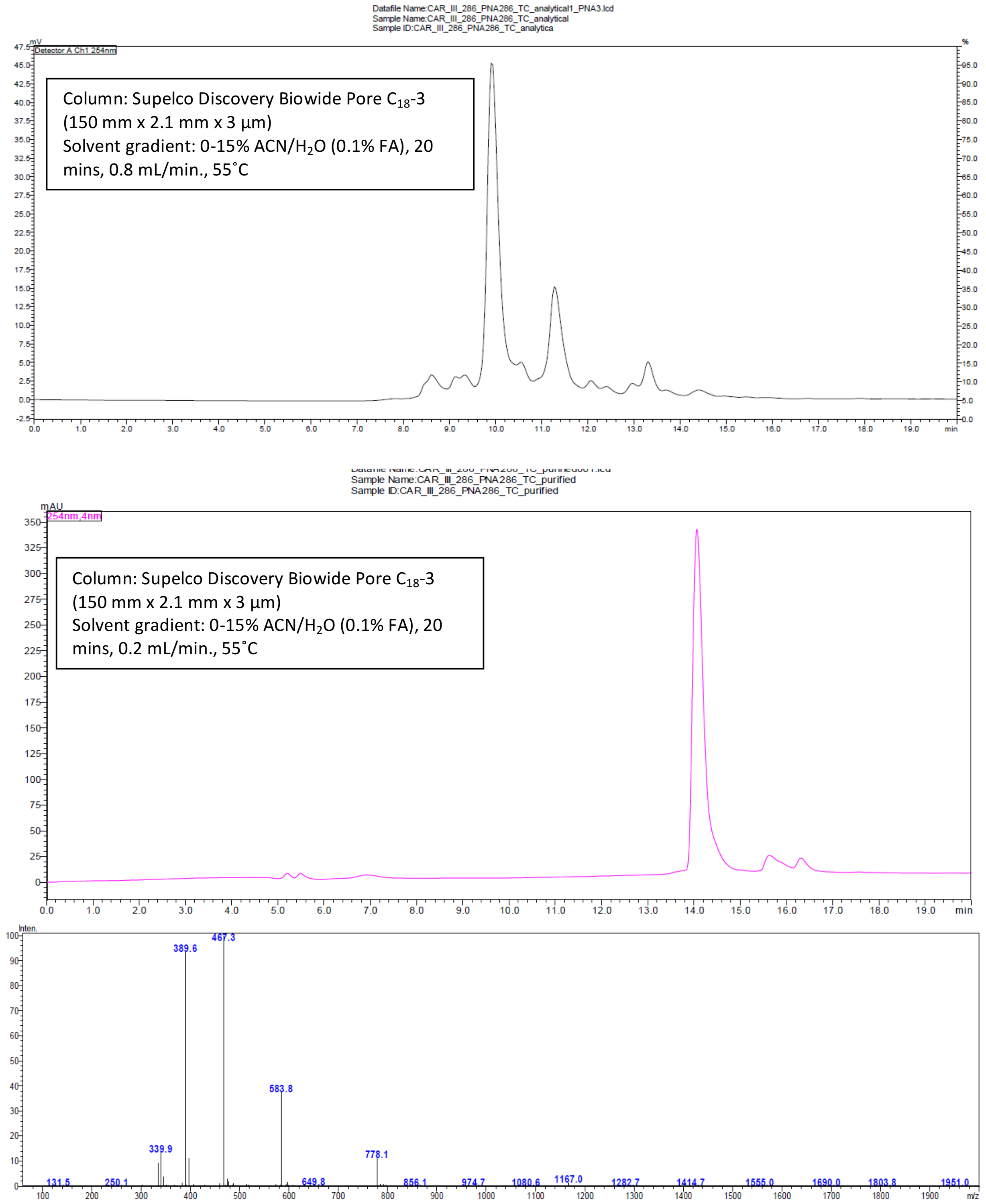

Figure S4. Crude HPLC (top) and purified LC/MS (middle/bottom) data of PNA1t.

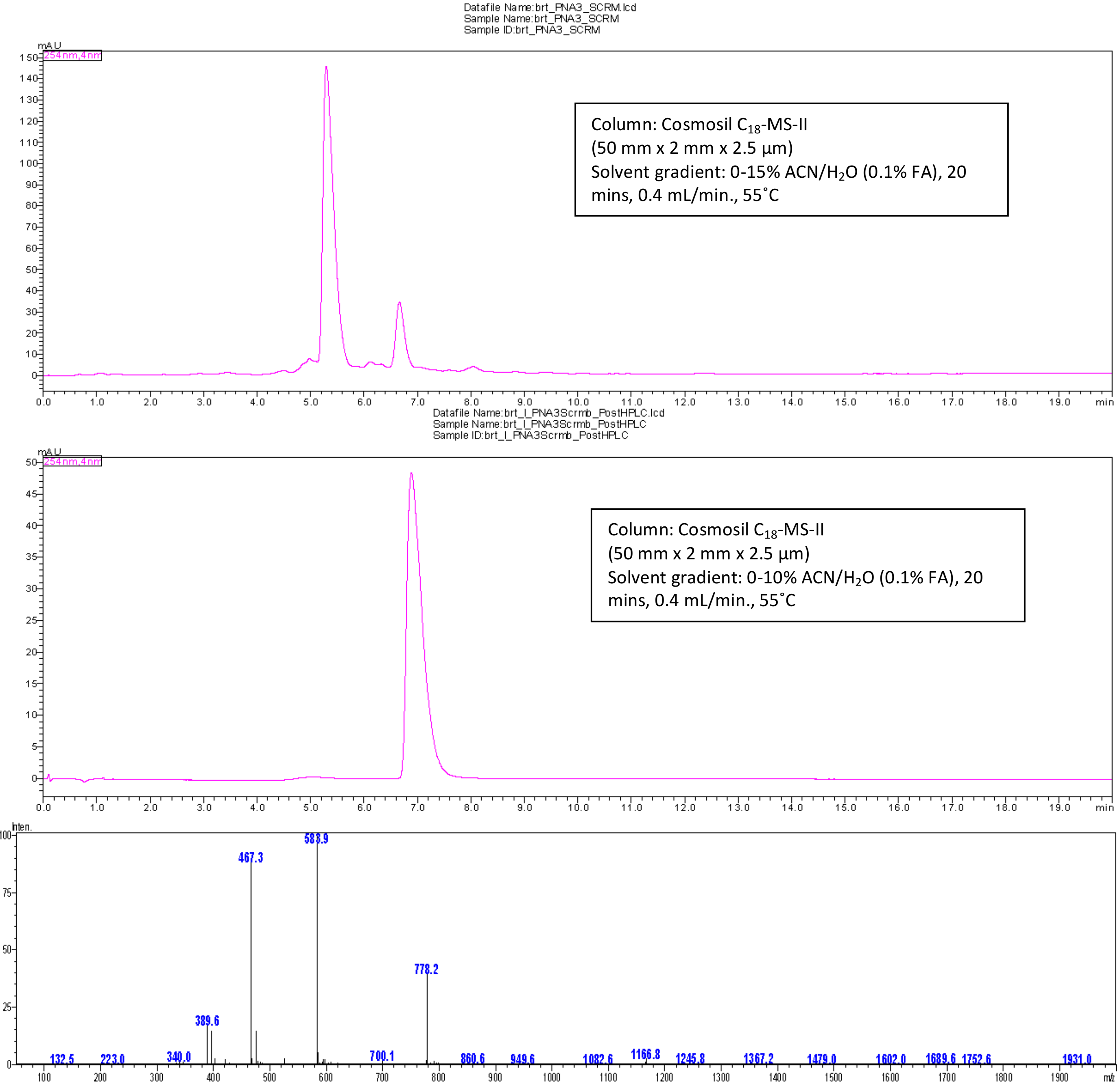

Figure S5. Crude HPLC (top) and purified LC/MS (middle/bottom) data of PNA1ts.

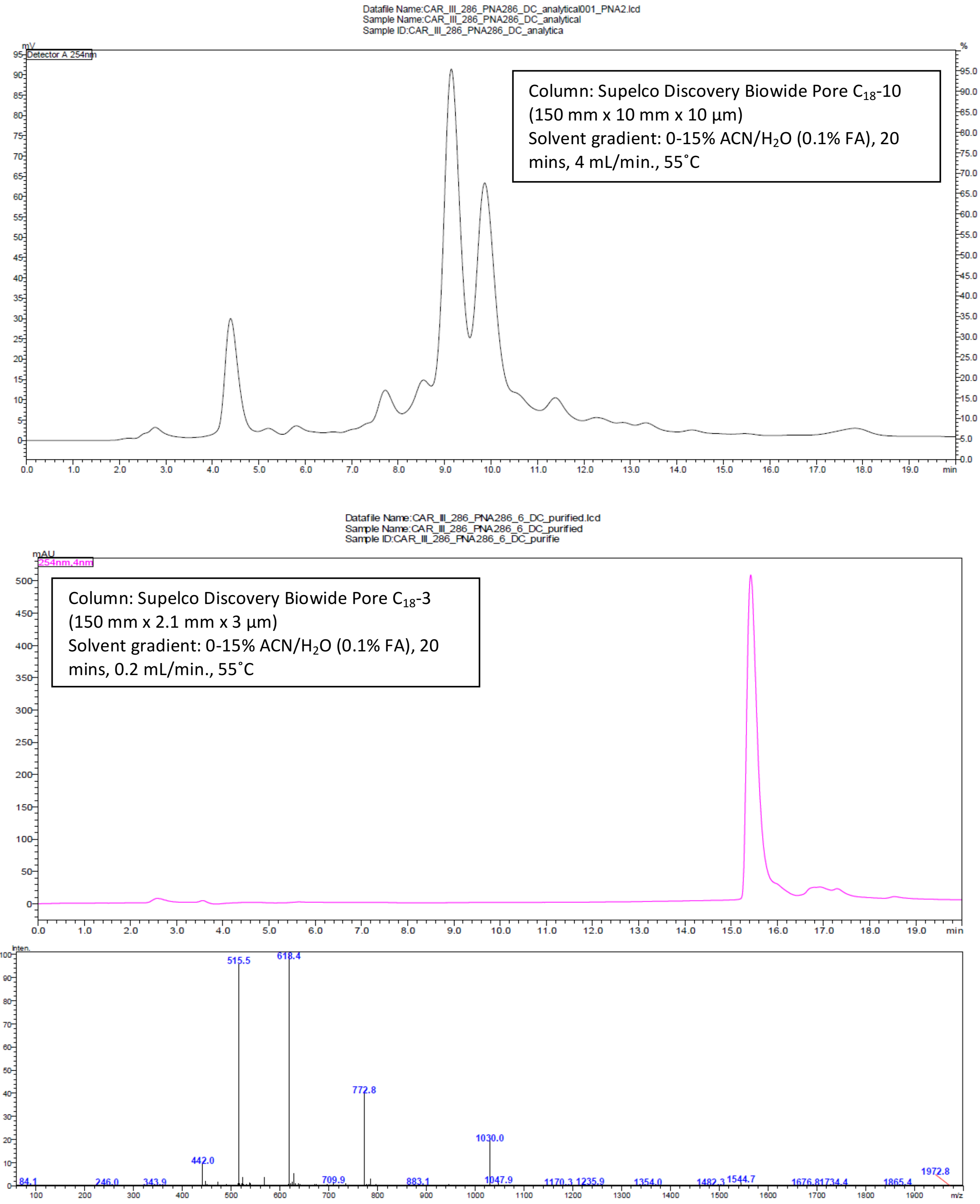

Figure S6. Crude HPLC (top) and purified LC/MS (middle/bottom) data of PNA1d.

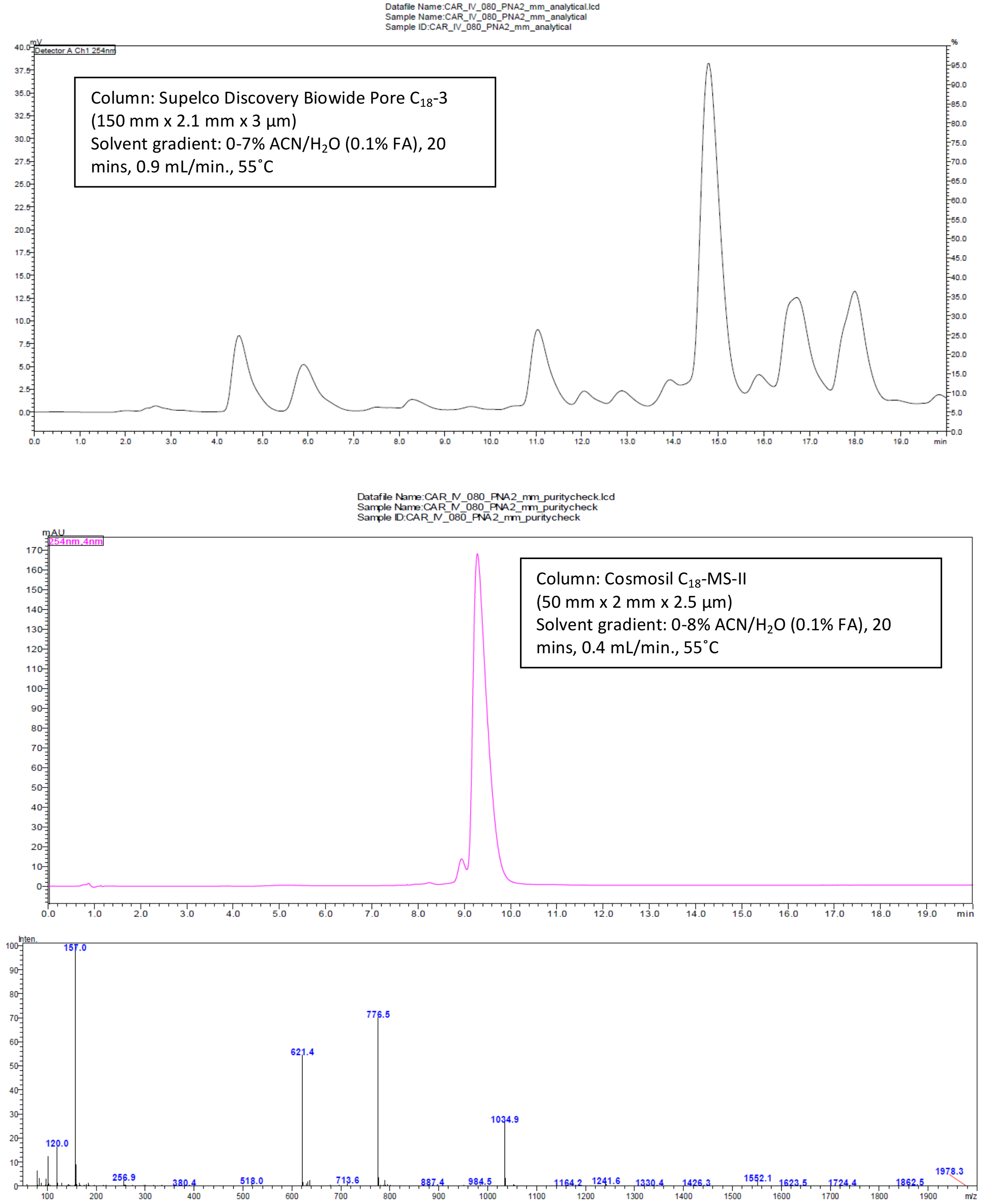

Figure S7. Crude HPLC (top) and purified LC/MS (middle/bottom) data of PNA1dm.

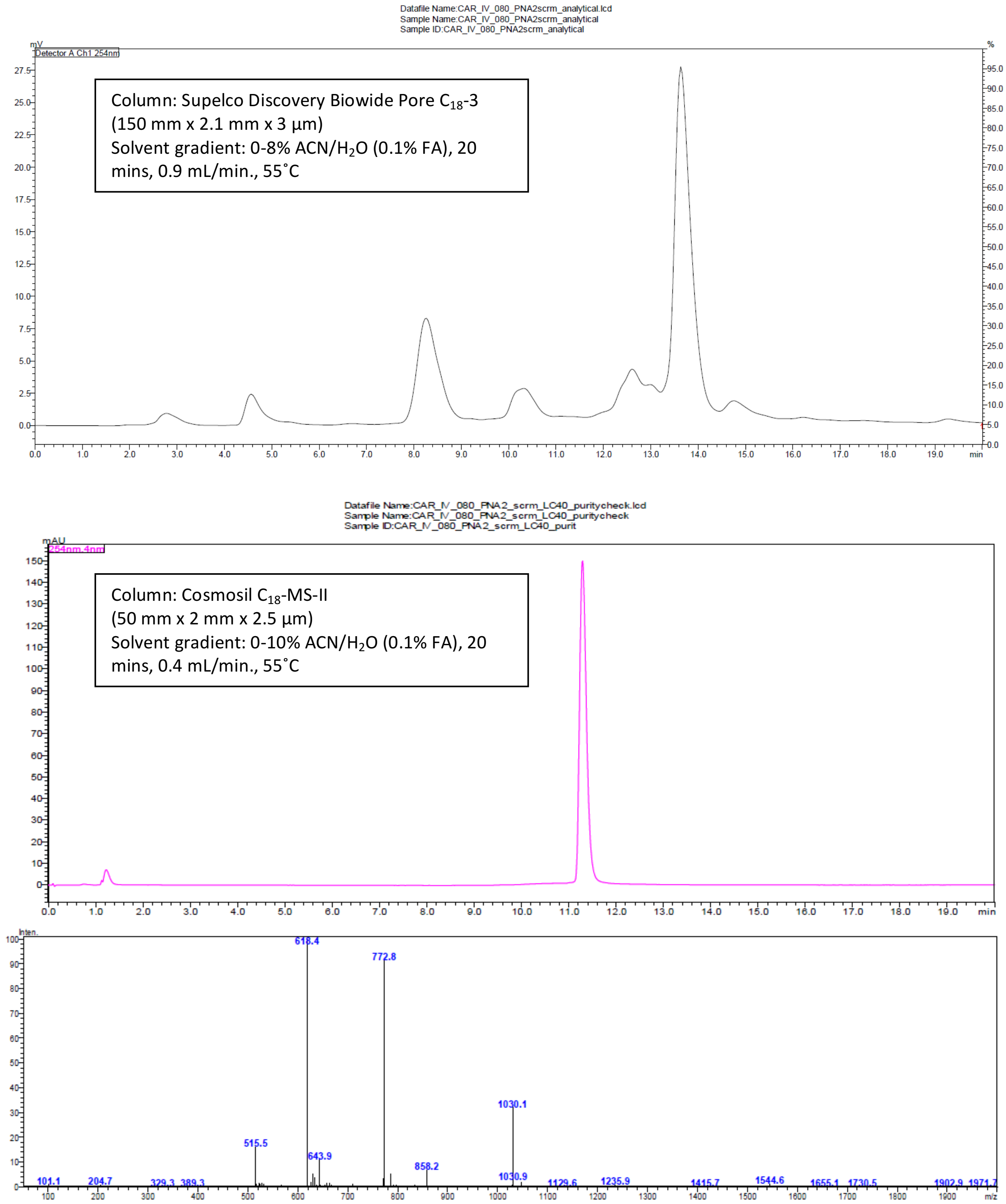

Figure S8. Crude HPLC (top) and purified LC/MS (middle/bottom) data of PNA1ds.

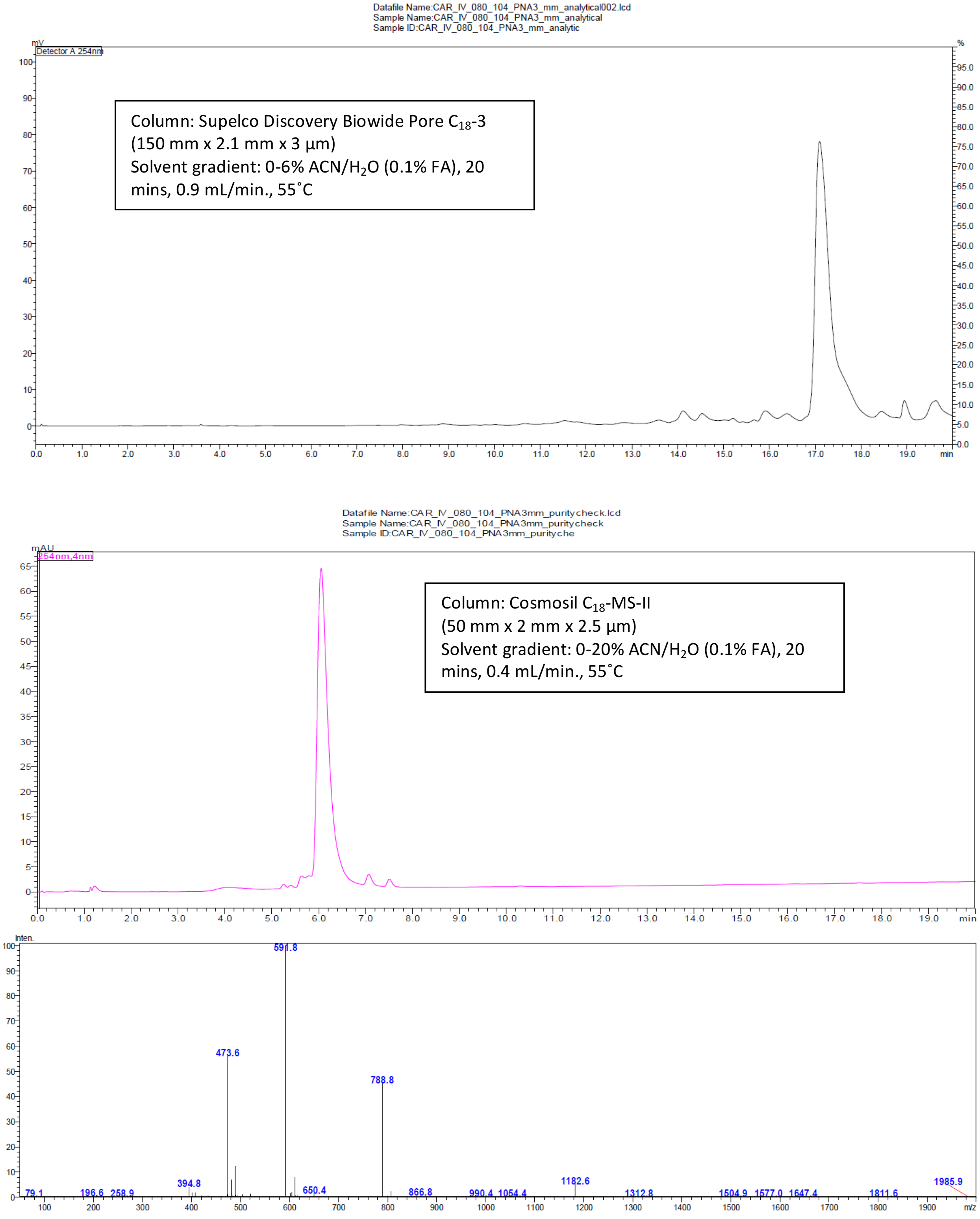

Figure S9. Crude HPLC (top) and purified LC/MS (middle/bottom) data of PNA1tm.

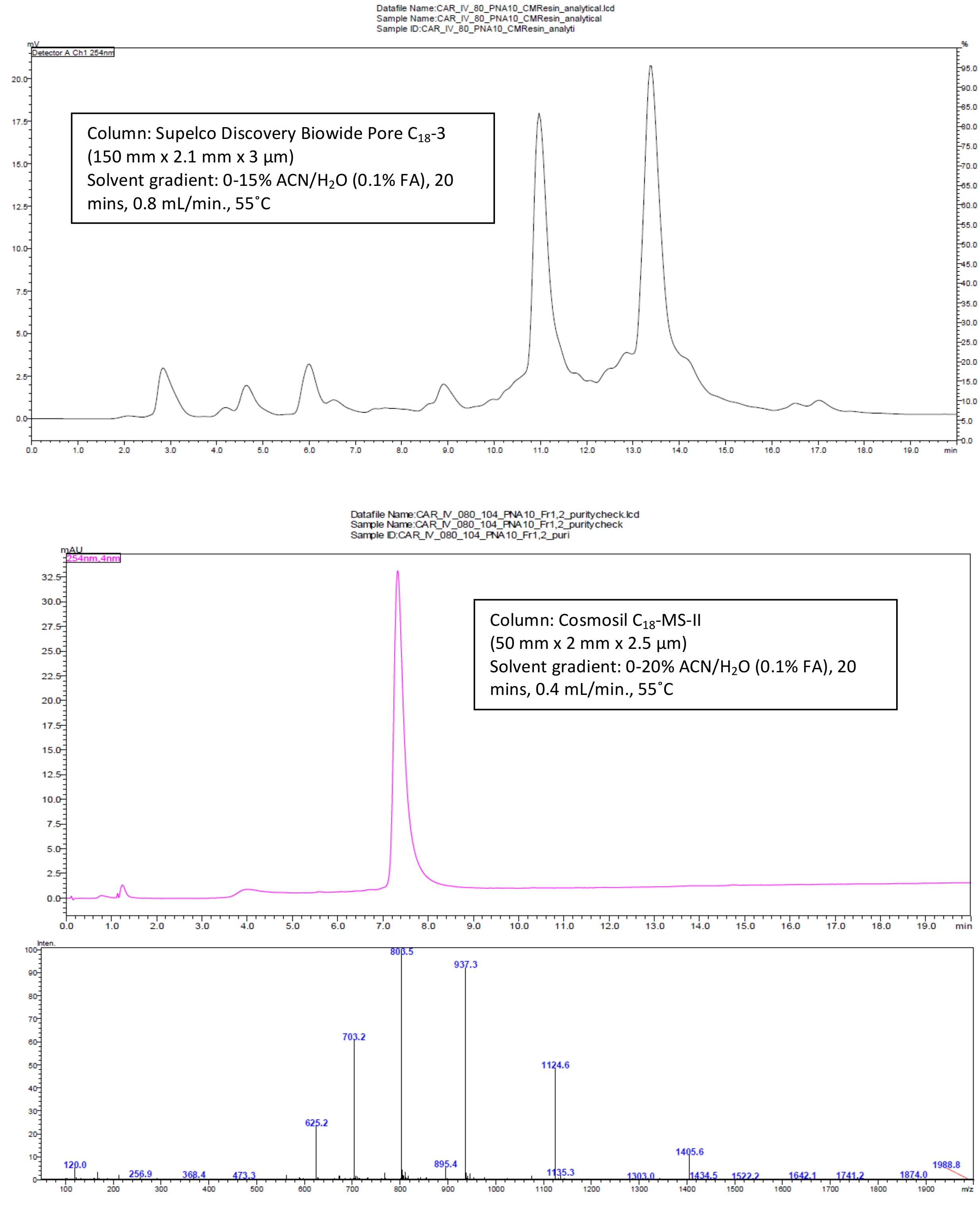

Figure S10. Crude HPLC (top) and purified LC/MS (middle/bottom) data of PNA2.

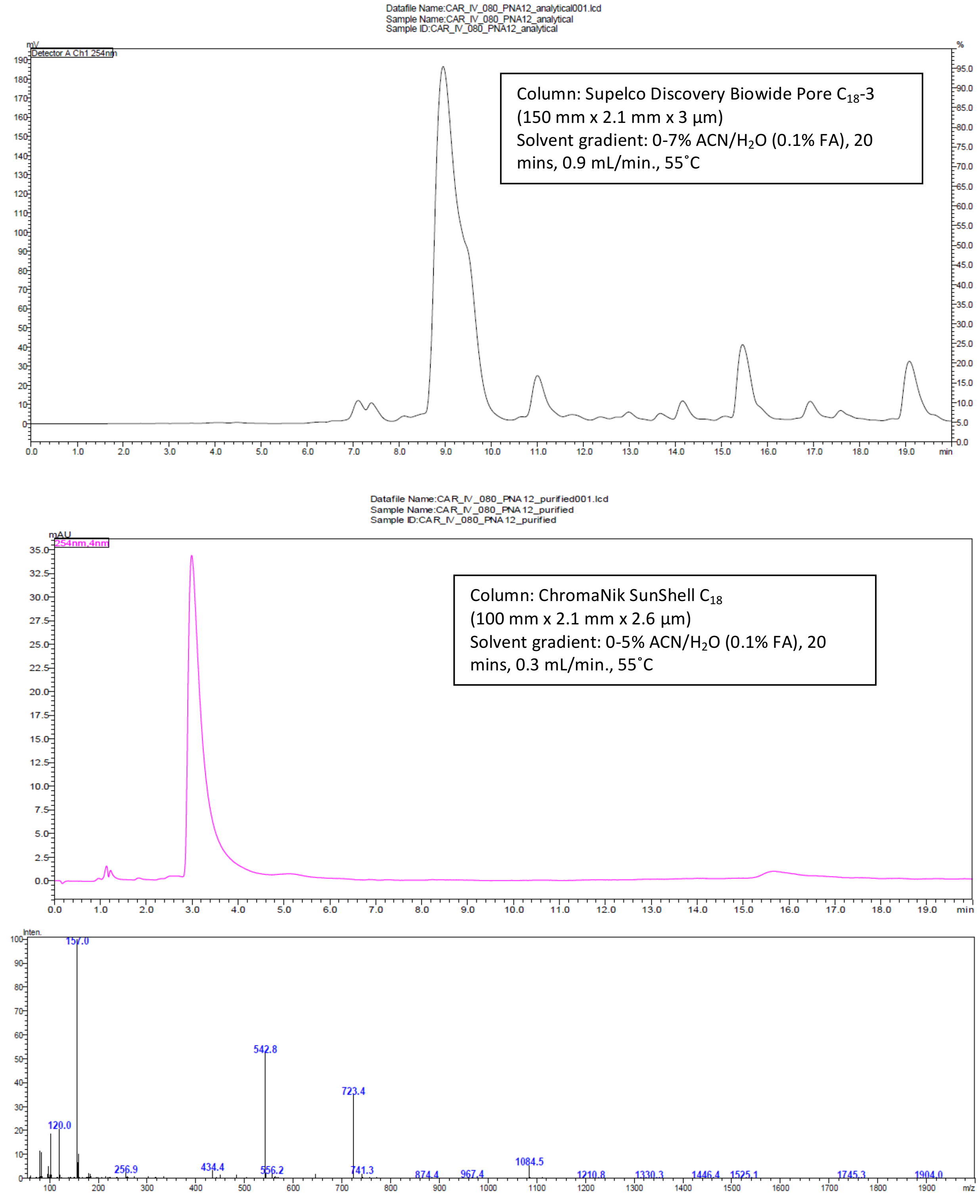

Figure S11. Crude HPLC (top) and purified LC/MS (middle/bottom) data of PNA2t.

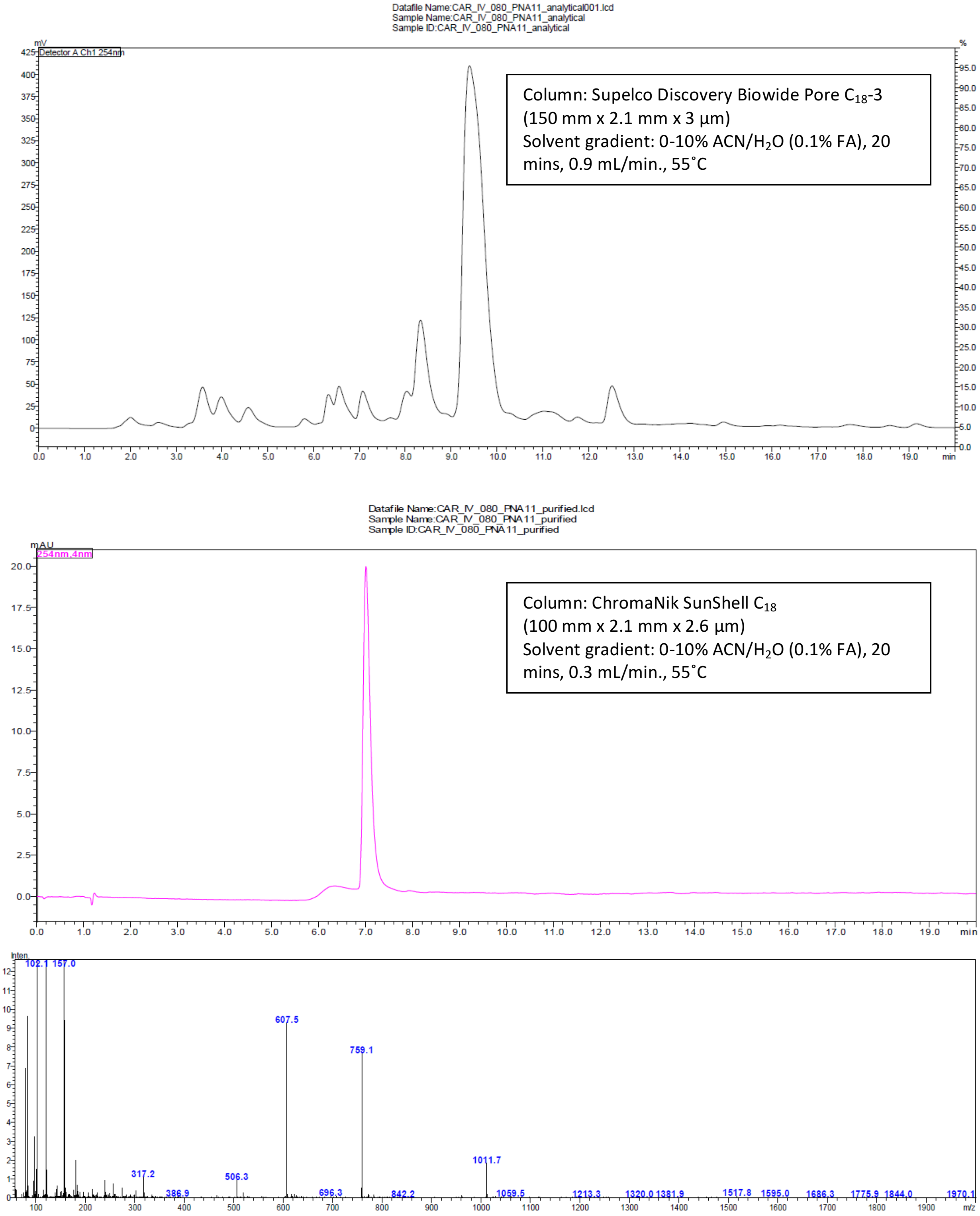

Figure S12. Crude HPLC (top) and purified LC/MS (middle/bottom) data of PNA2d.

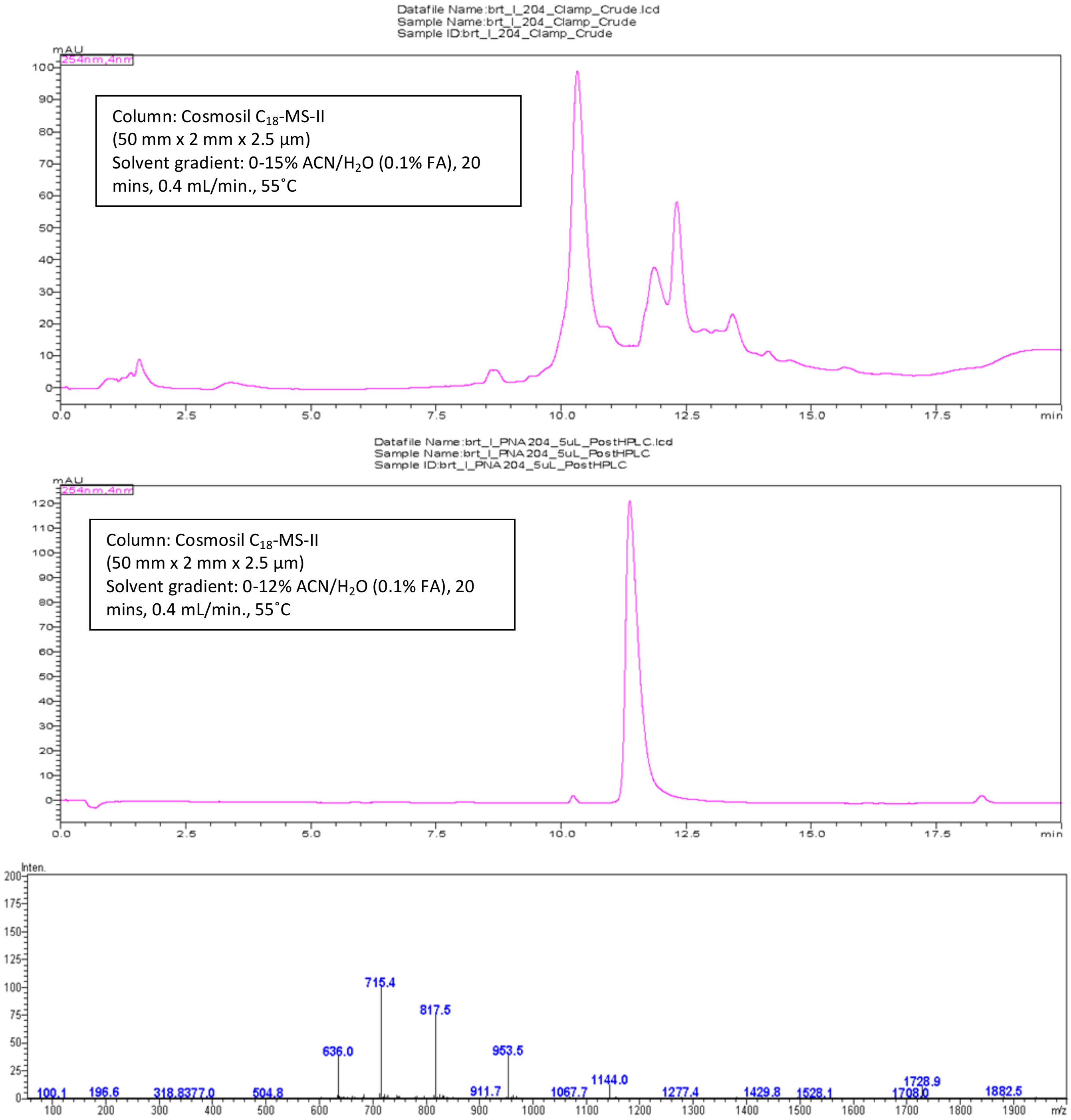

Figure S13. Crude HPLC (top) and purified LC/MS (middle/bottom) data of PNA3.

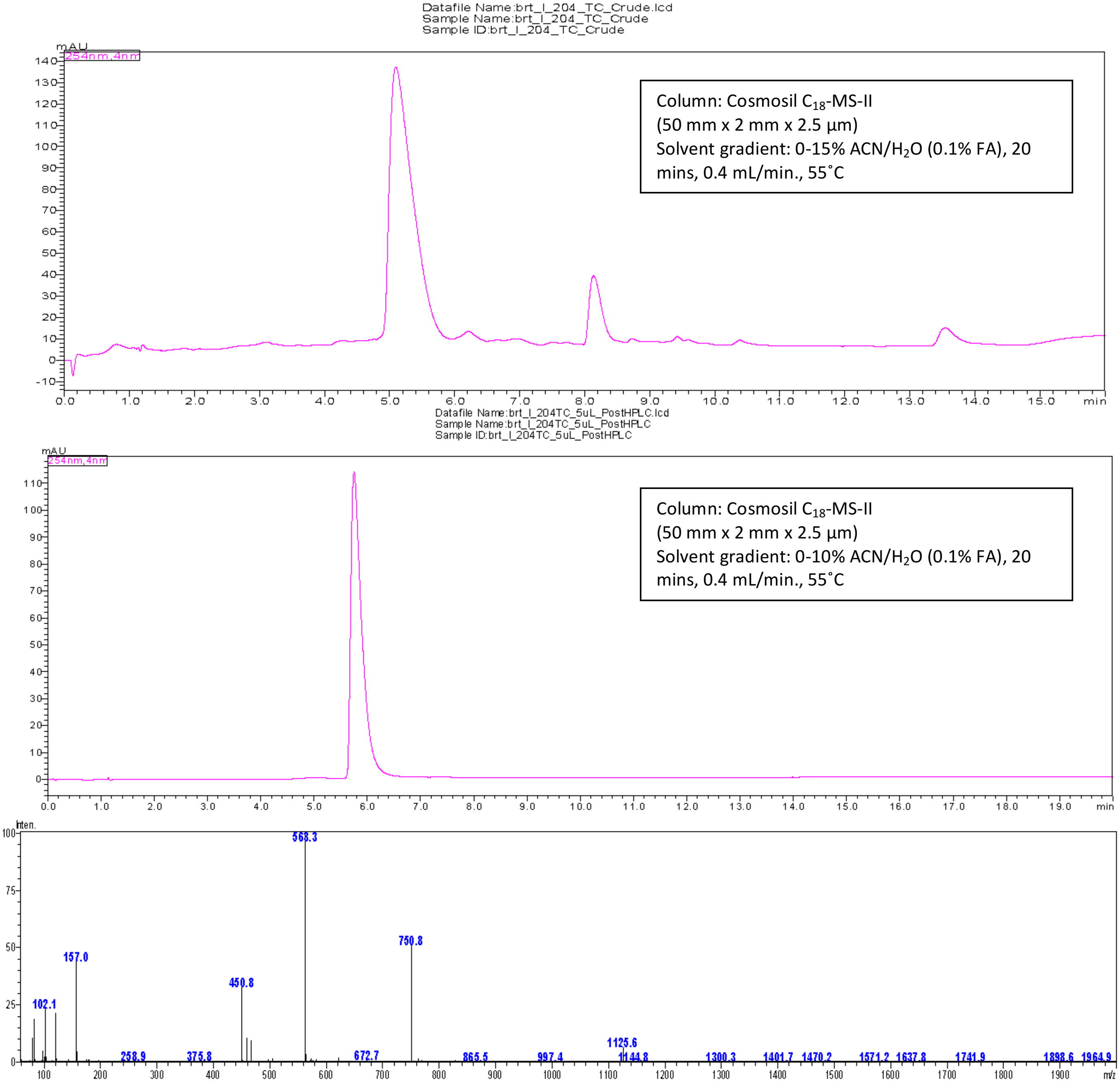

Figure S14. Crude HPLC (top) and purified LC/MS (middle/bottom) data of PNA3t.

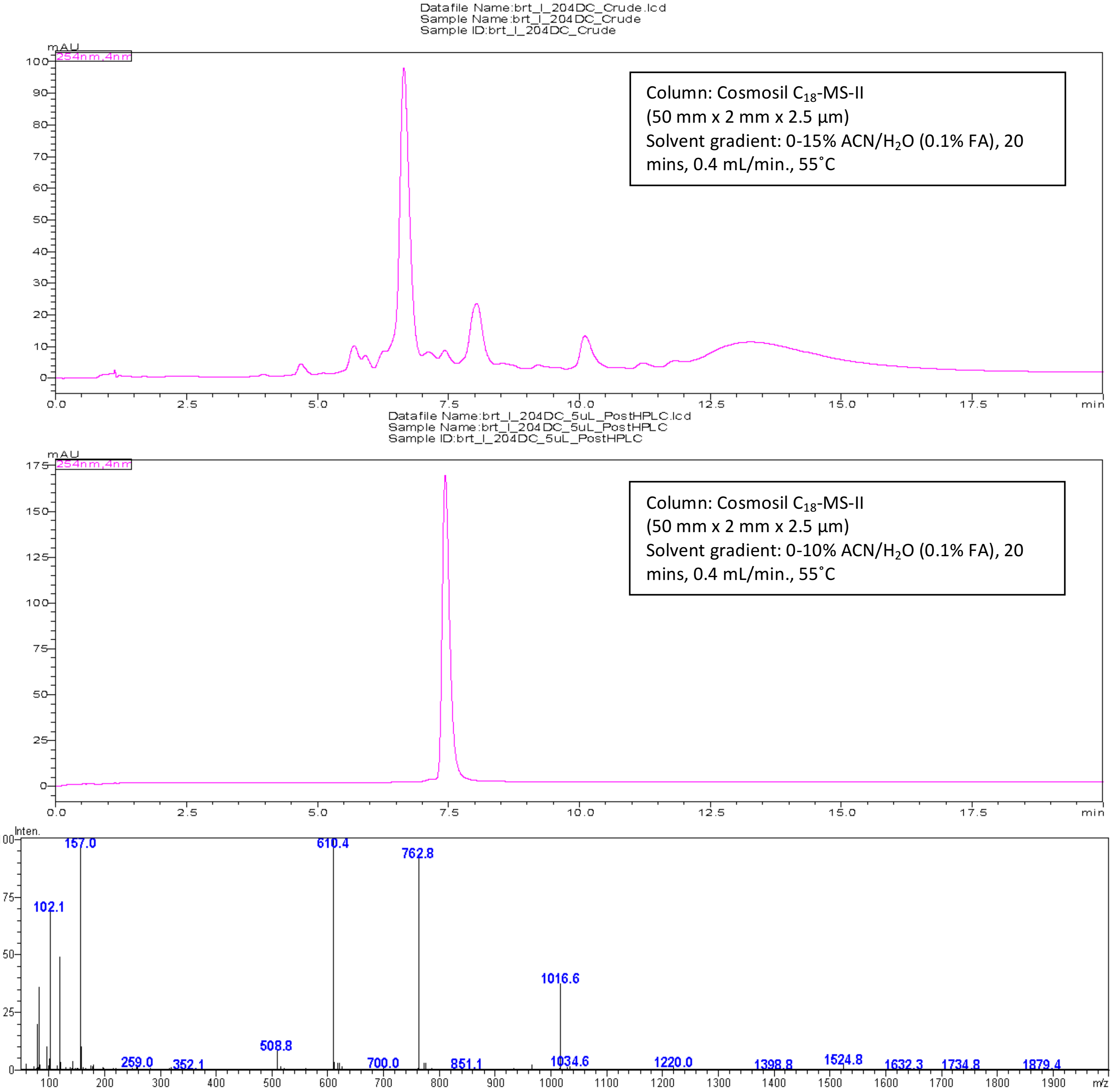

Figure S15. Crude HPLC (top) and purified LC/MS (middle/bottom) data of PNA3d.

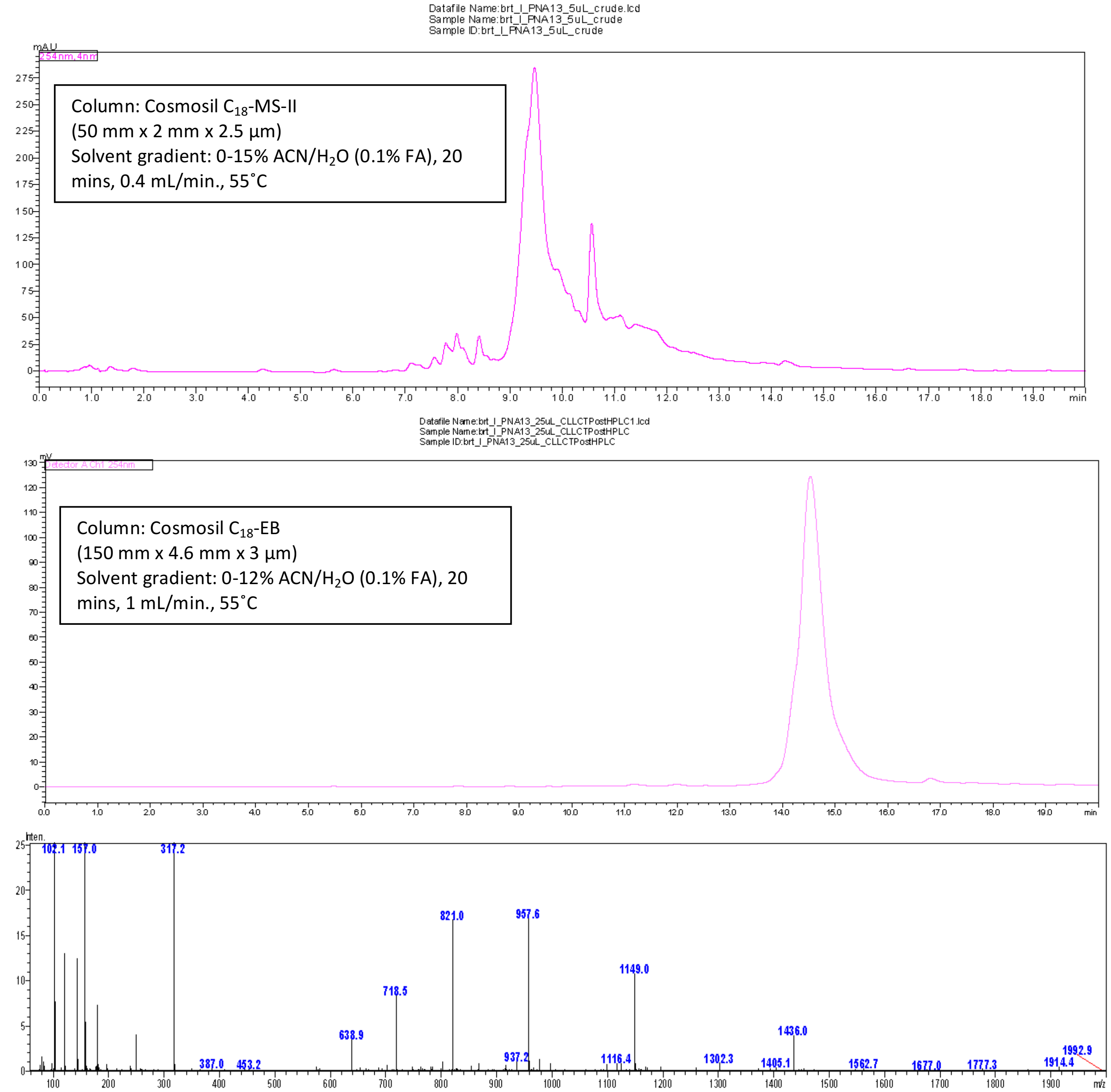

Figure S16. Crude HPLC (top) and purified LC/MS (middle/bottom) data of PNA4.

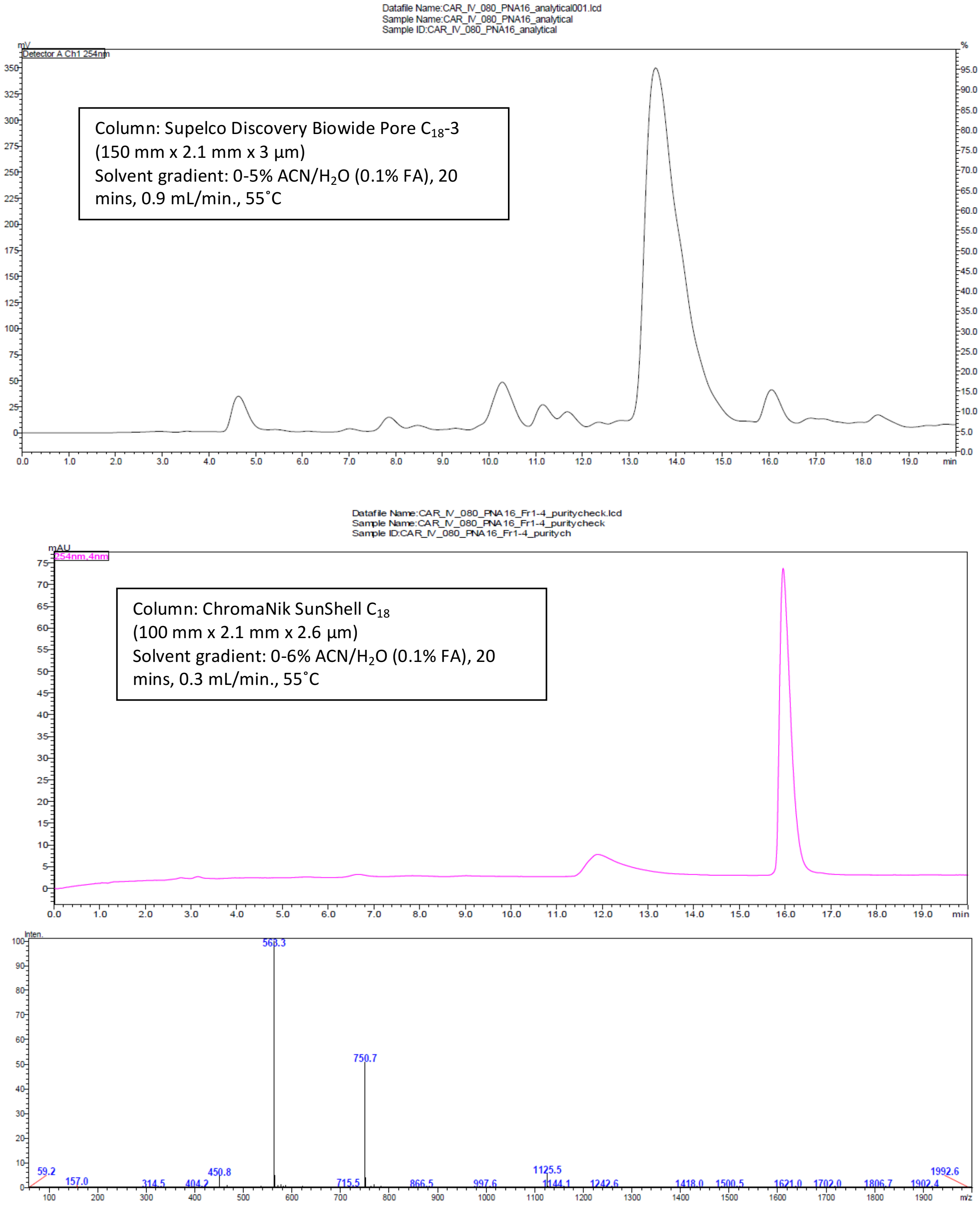

Figure S17. Crude HPLC (top) and purified LC/MS (middle/bottom) data of PNA4t.

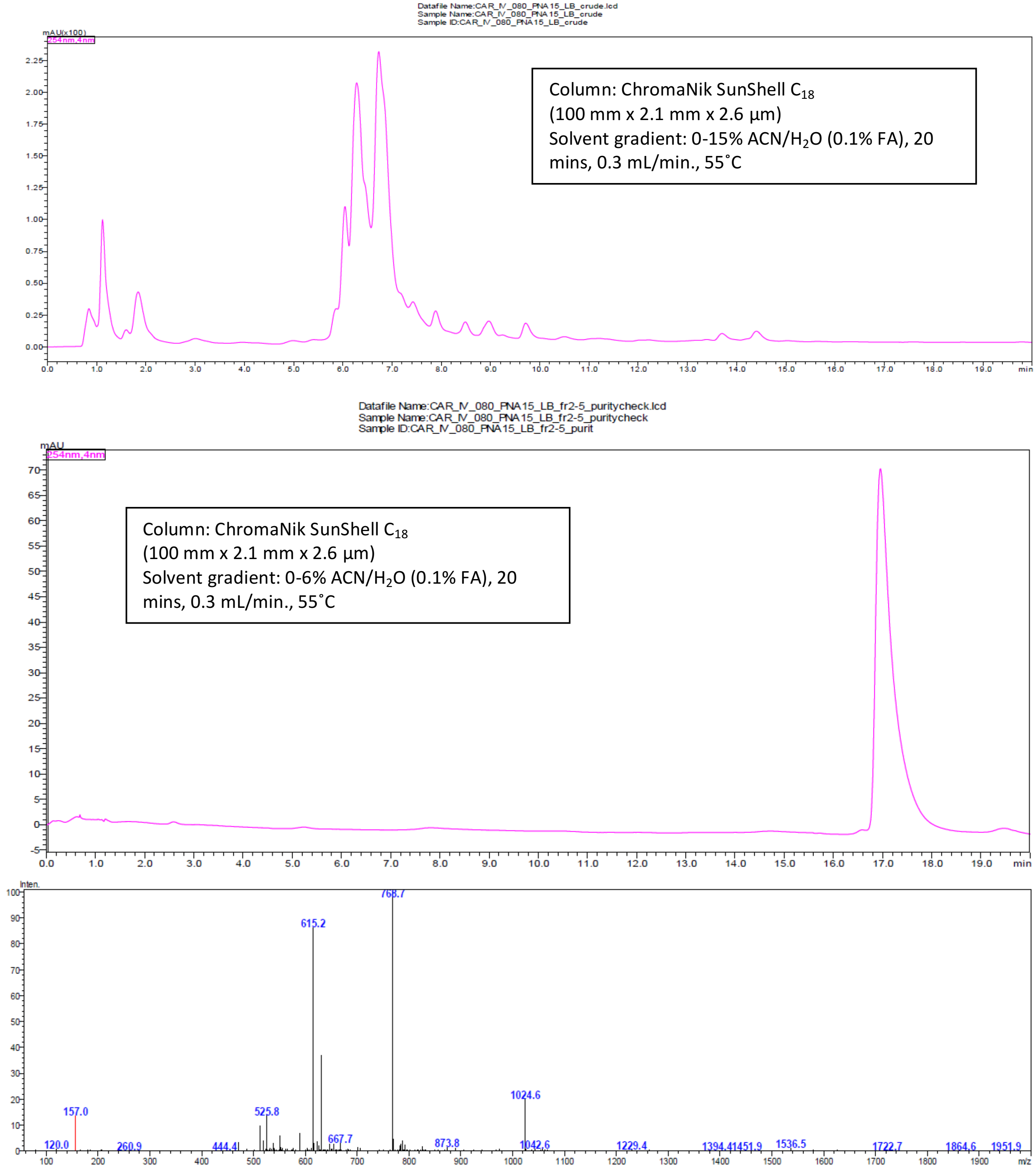

Figure S18. Crude HPLC (top) and purified LC/MS (middle/bottom) data of PNA4d.

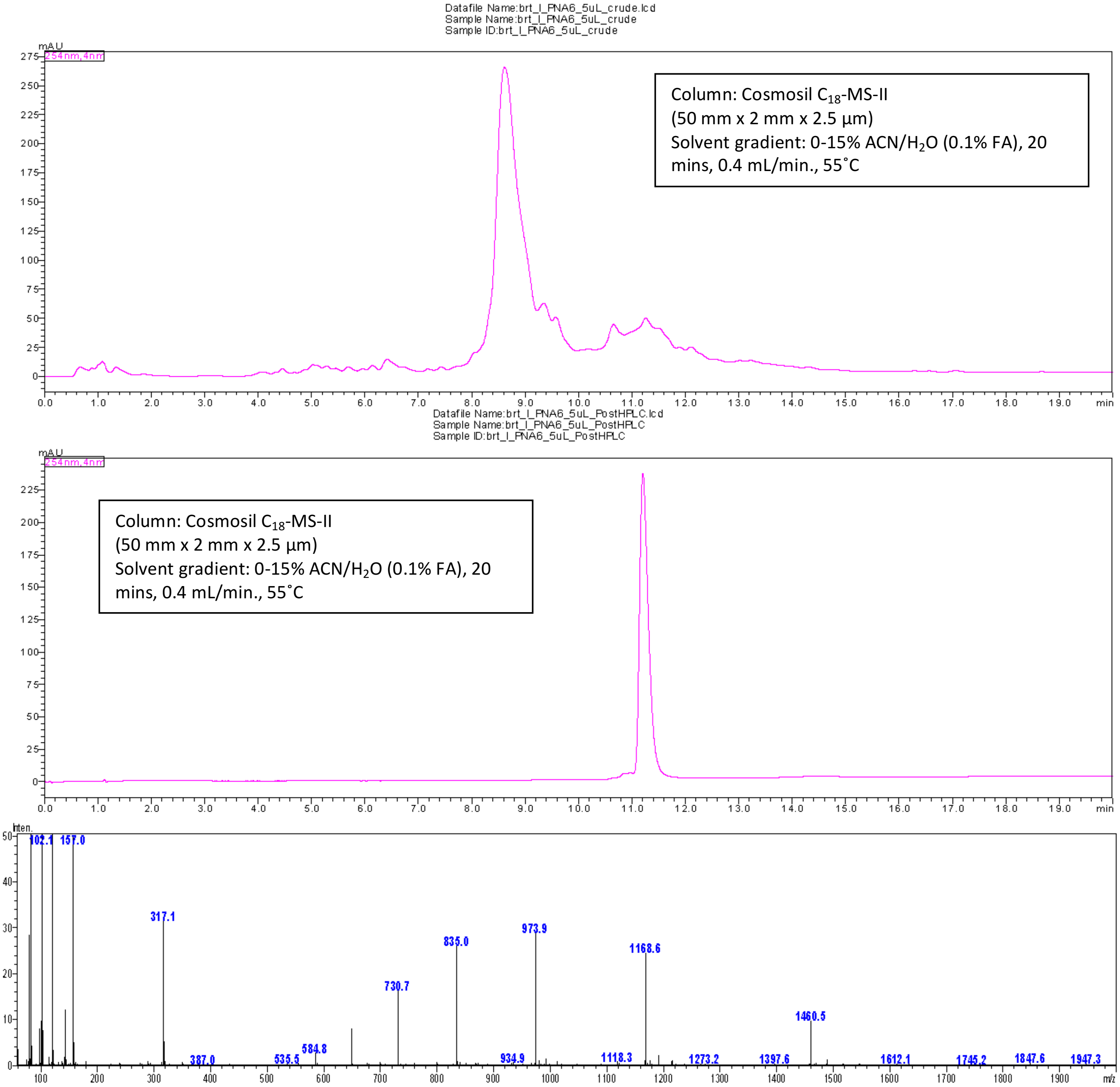

Figure S19. Crude HPLC (top) and purified LC/MS (middle/bottom) data of PNA5.

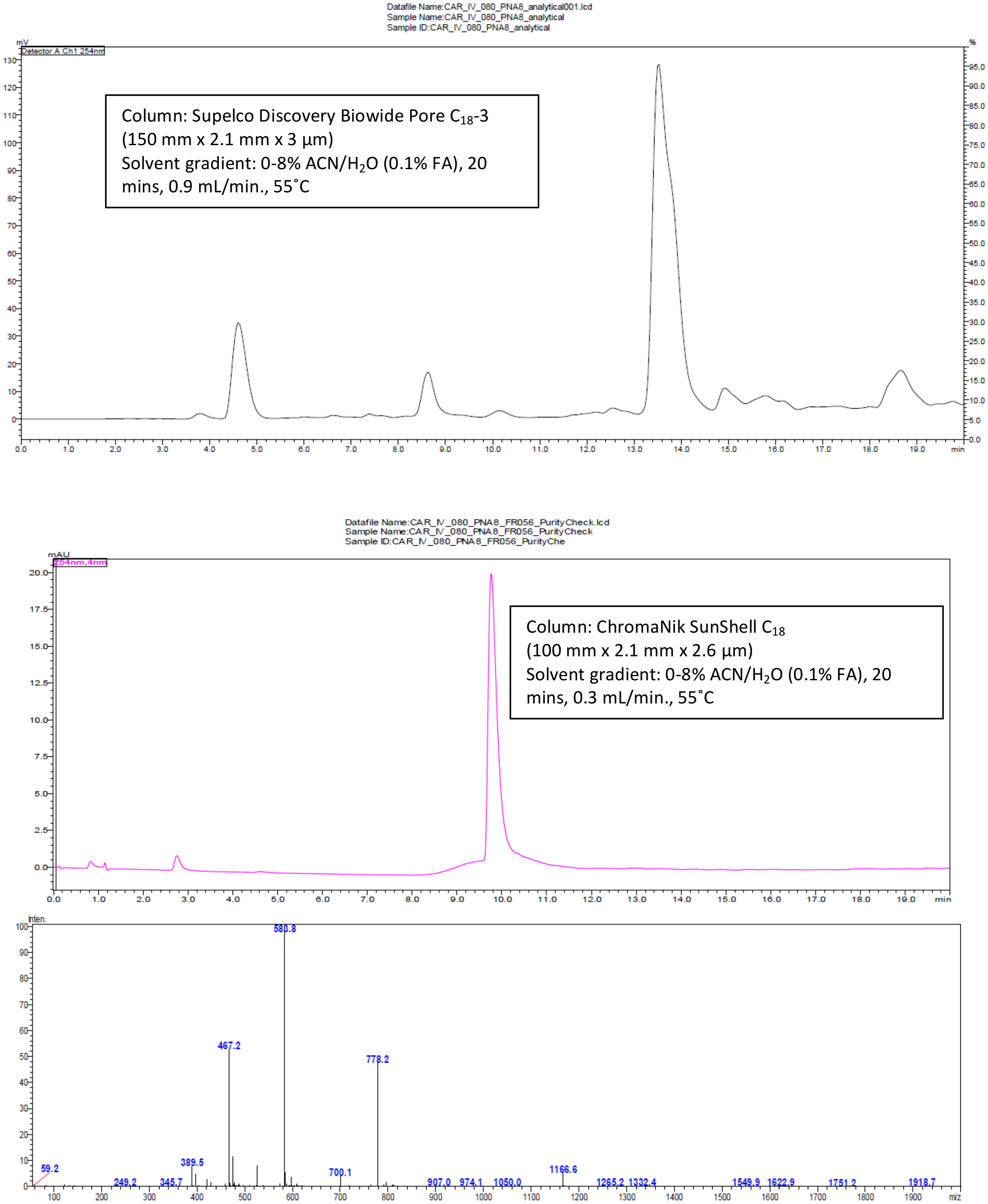

Figure S20. Crude HPLC (top) and purified LC/MS (middle/bottom) data of PNA5t.

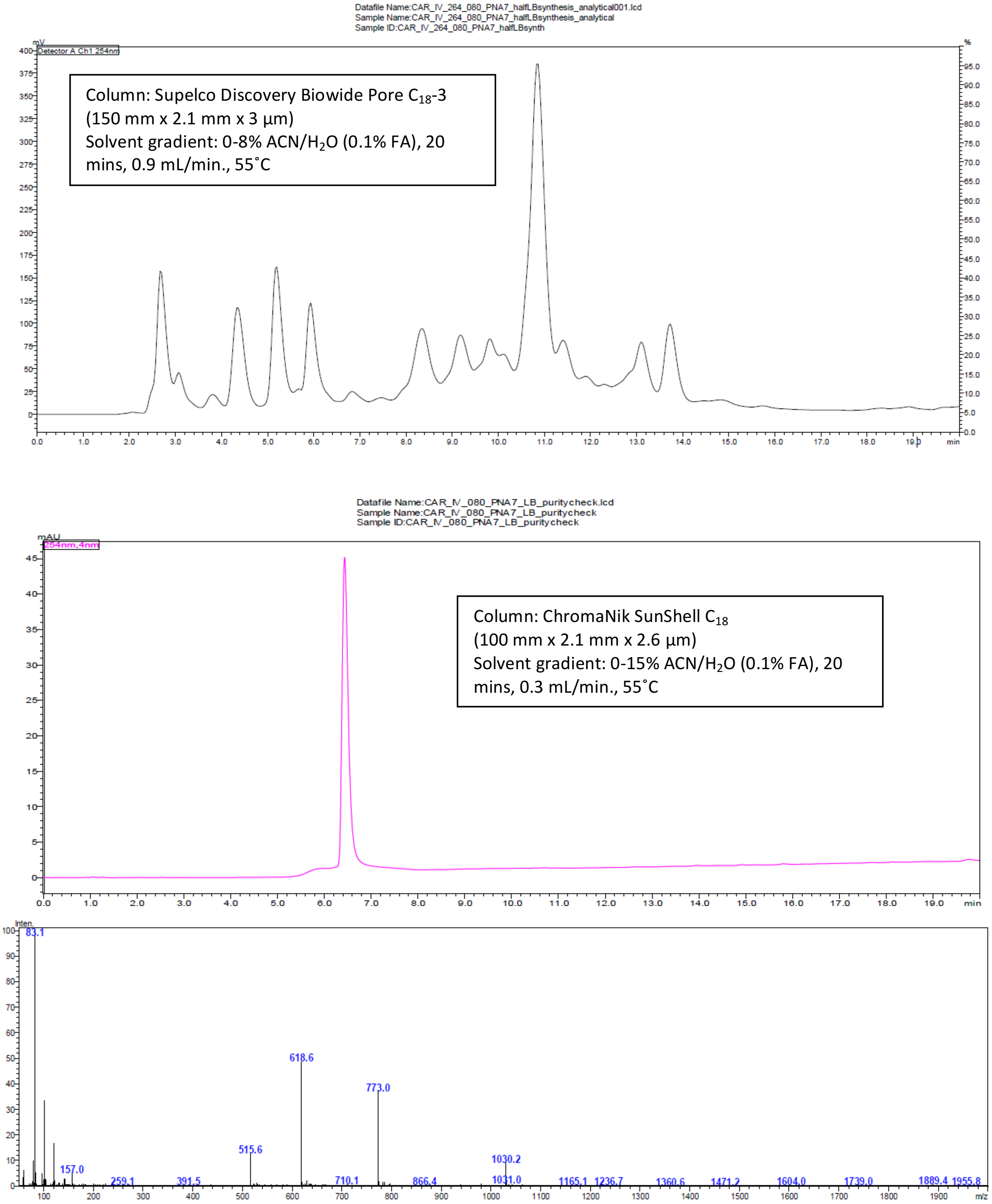

Figure S21. Crude HPLC (top) and purified LC/MS (middle/bottom) data of PNA5d.

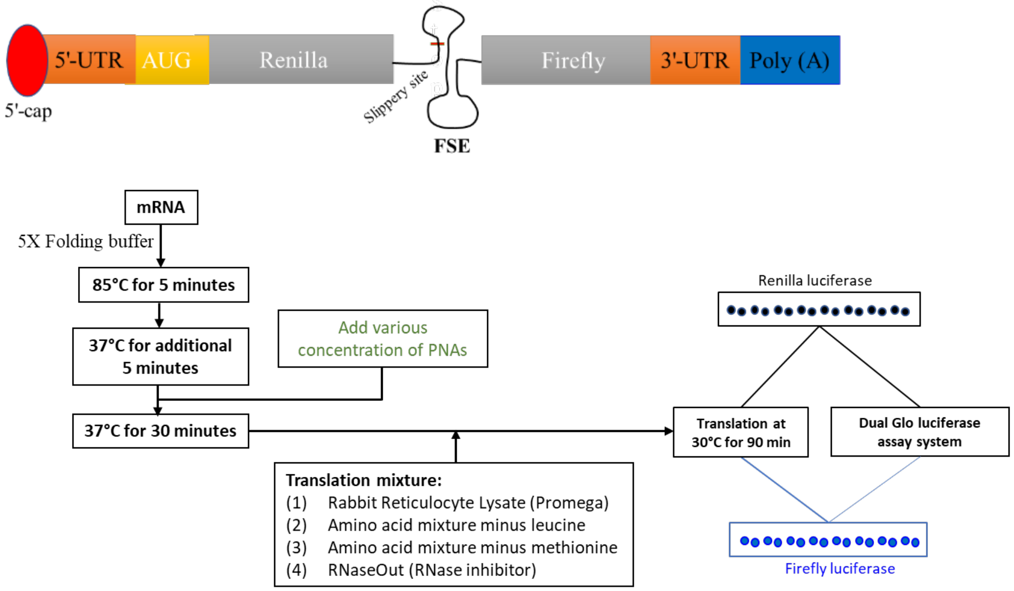

Figure S22. Workflow of the dual luciferase assay.

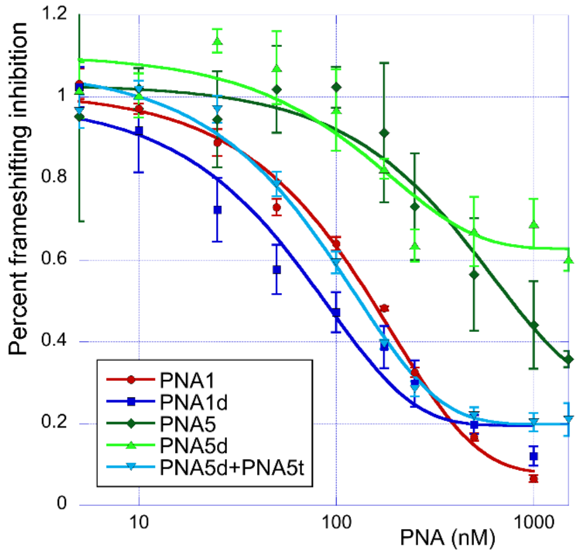

Figure S23. Comparison of normalized inhibition of frameshifting by PNA1 targeting stem 2 and PNA5 targeting stem 3 of SARS-CoV-2 FSE.

PNA1

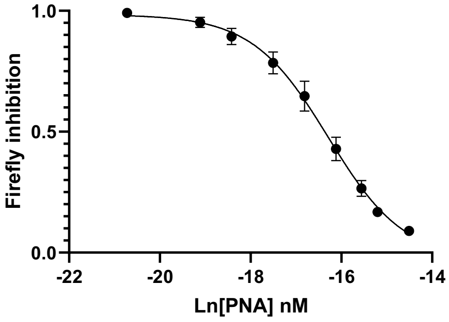

PNA1m

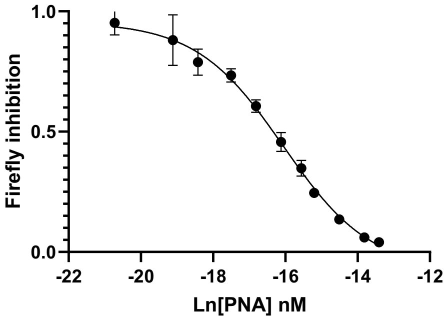

PNA1d

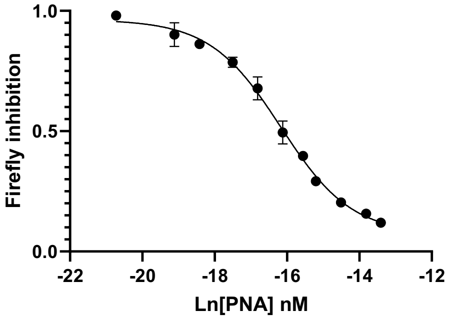

PNA1dm

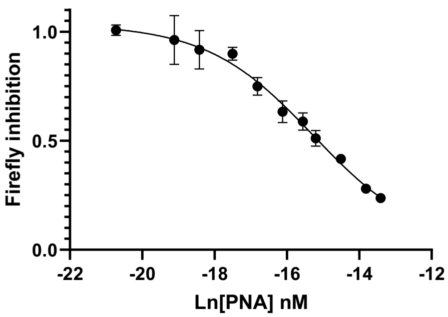

PNA1d + PNA1t

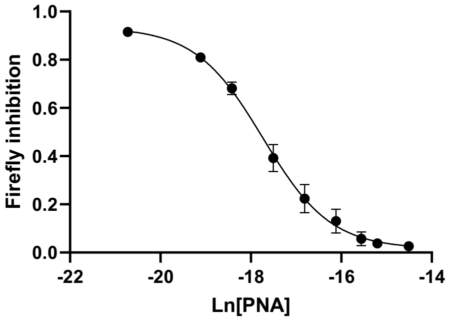

PNA1d + PNA1ts

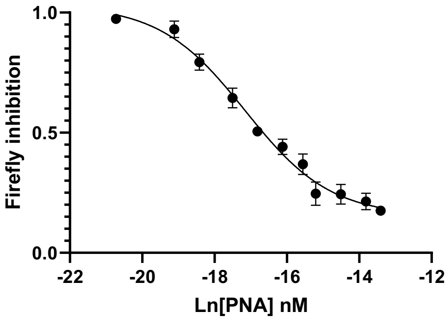

PNA1d + PNA1tm

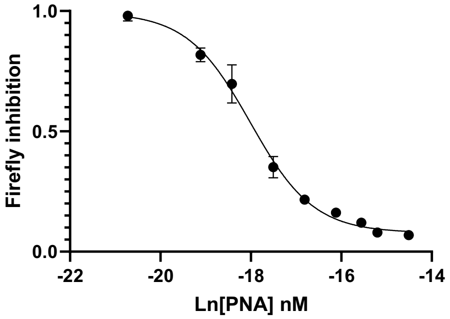

PNA1dm + PNA1t

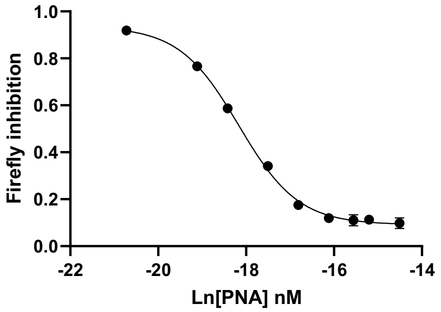

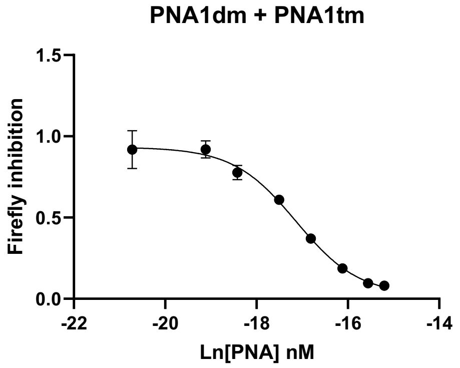

Figure S24. Results of the dual luciferase assay of PNA1 and control PNAs (Table 1 and Figure 3) targeting SARS-CoV-2 FSE RNA.

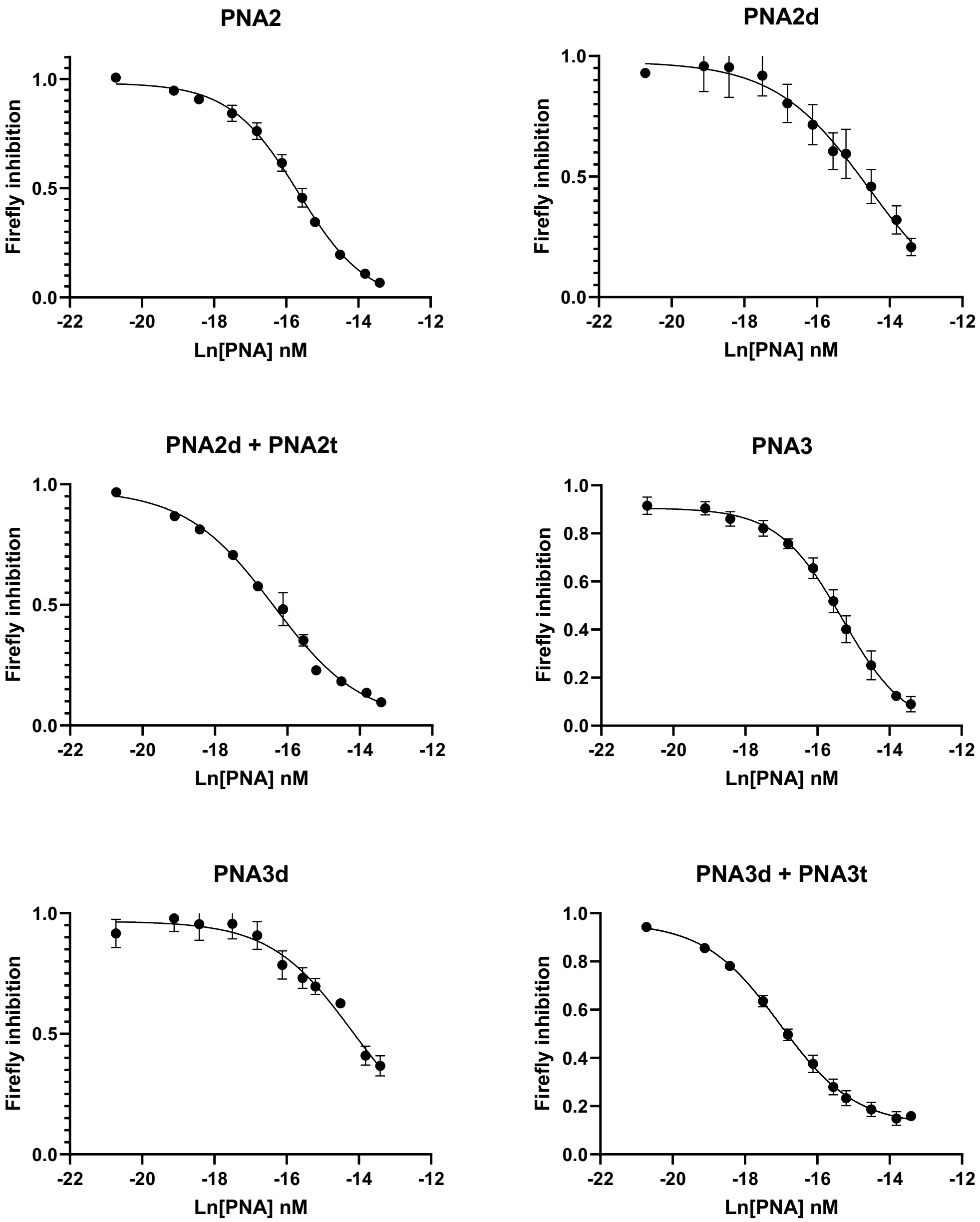

Figure S25. Results of the dual luciferase assay of PNA2, PNA 3 and control PNAs (Table 2) targeting SARS-CoV-2 FSE RNA.

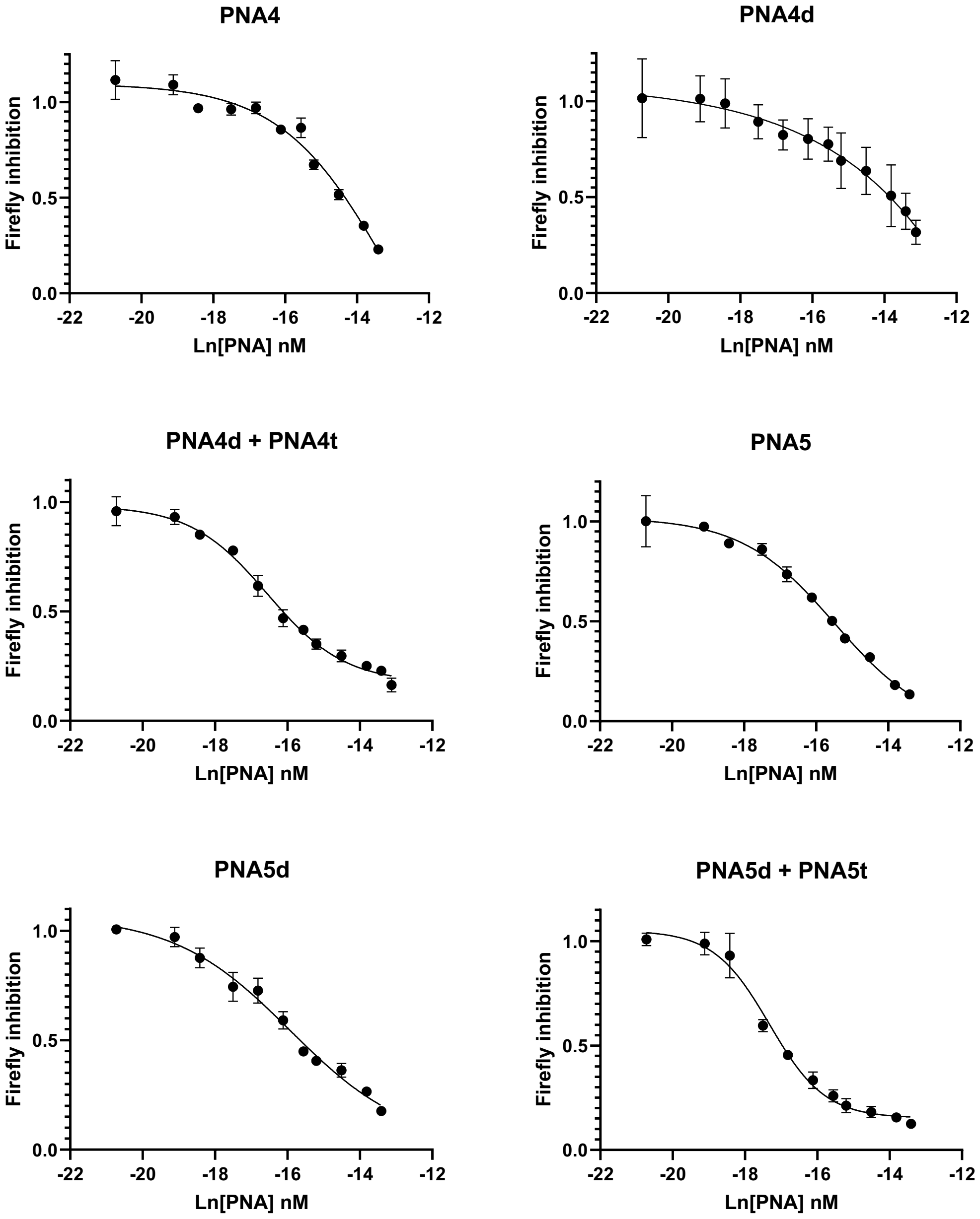

Figure S26. Results of the dual luciferase assay of PNA4, PNA 5 and control PNAs (Table 2) targeting SARS-CoV-2 FSE RNA.

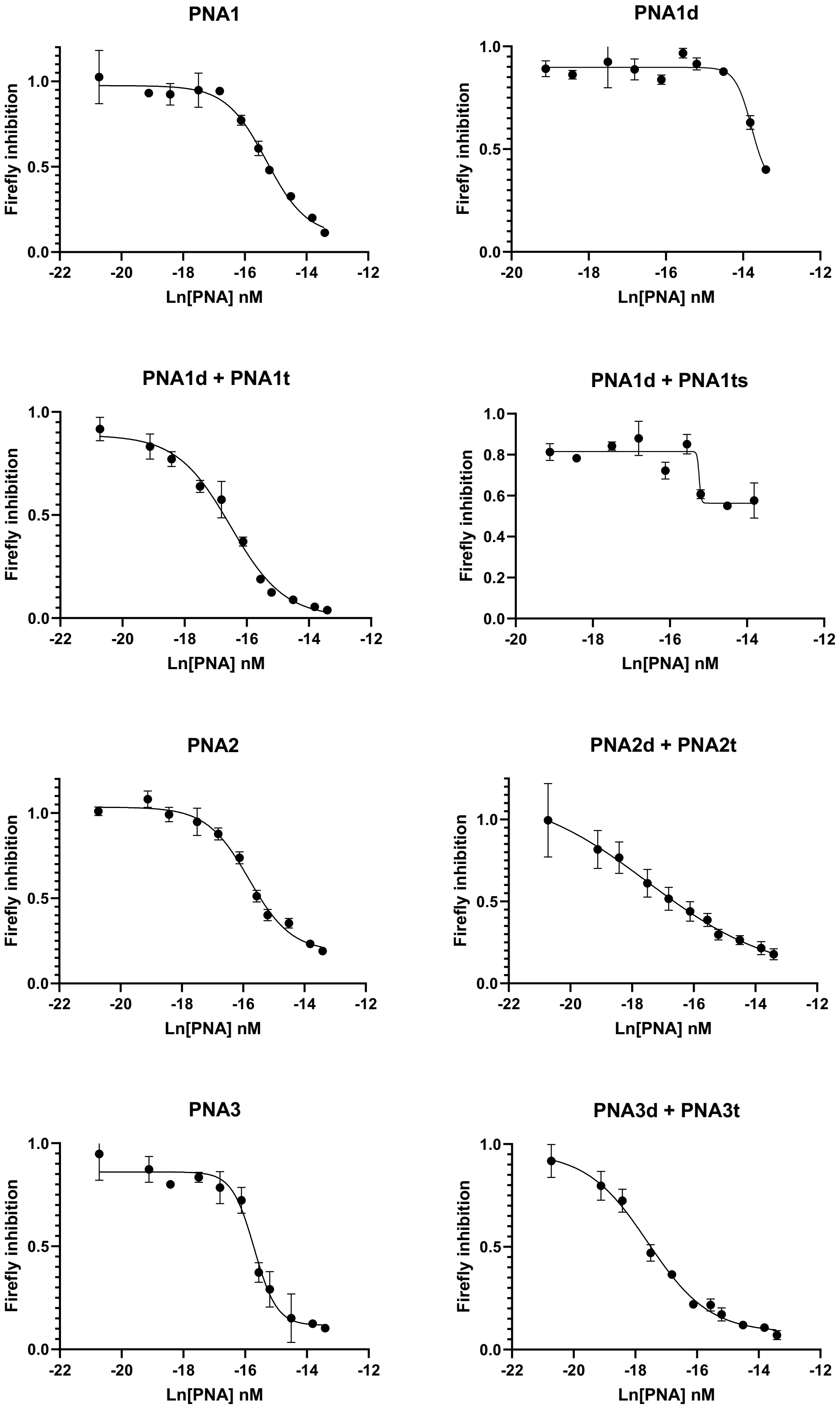
PNA1
PNA1d
PNA1d + PNA1t
PNA1d + PNA1ts
PNA2
PNA2d + PNA2t
PNA3
PNA3d + PNA3t
Firefly inhibition
Ln[PNA] nM

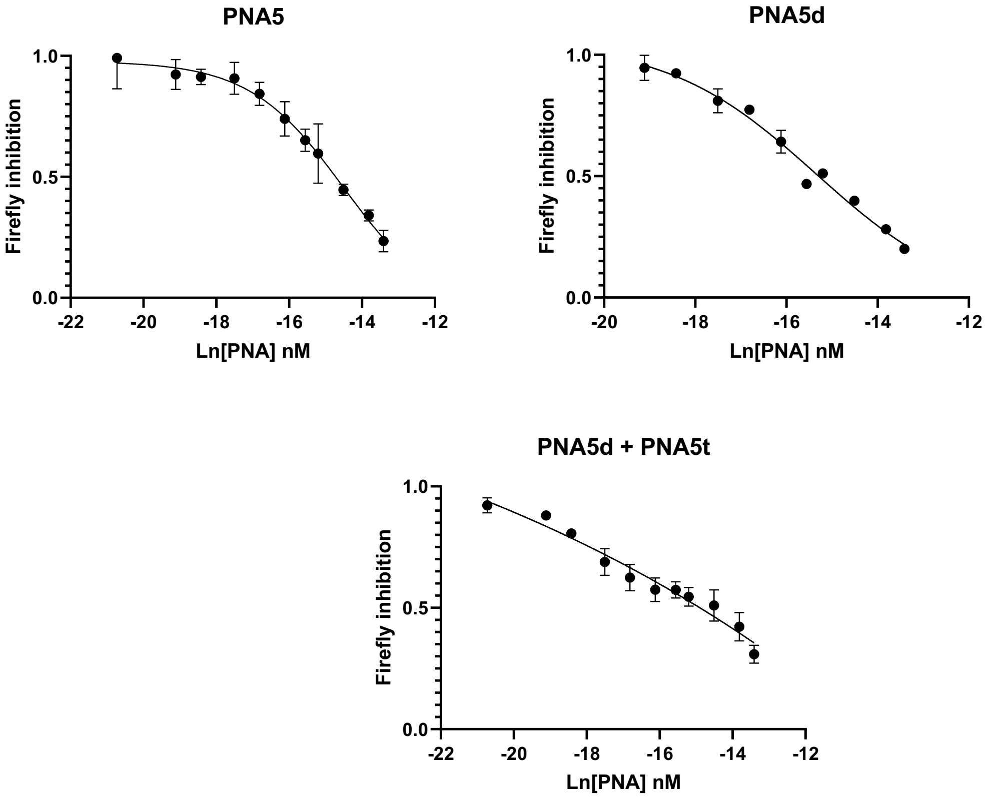

Figure S27. Results of the dual luciferase assay of PNAs targeting SARS-CoV-2 positive control (PC) RNA.

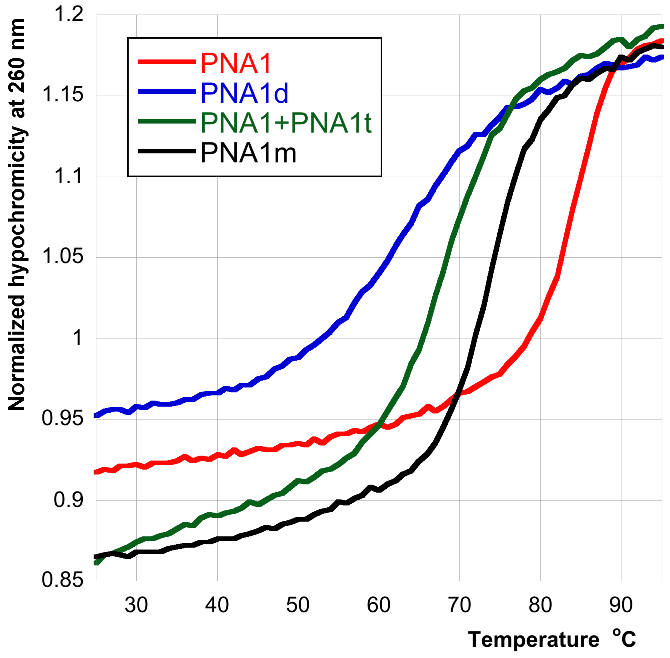

Figure S28. UV melting curves of PNA1 and control PNAs (Table 1) binding to model RNAs corresponding to stem 2.

Table S2. Melting data for PNAs against model RNAs corresponding to stem 2.[a]

	PNA1	PNA1m	PNA1d	PNA1d + PNA1t
Melting Temperatures (°C)	83.6<br>83.4<br>83.7	73.5<br>73.3<br>73.1	65.9<br>65.2<br>65.2	69.9<br>69.3<br>69.7
Average<br>Std. Dev	83.6<br>0.2	73.3<br>0.2	65.5<br>0.4	69.6<br>0.3

[a] Melting measured at 260 nm against model ssRNA_S2

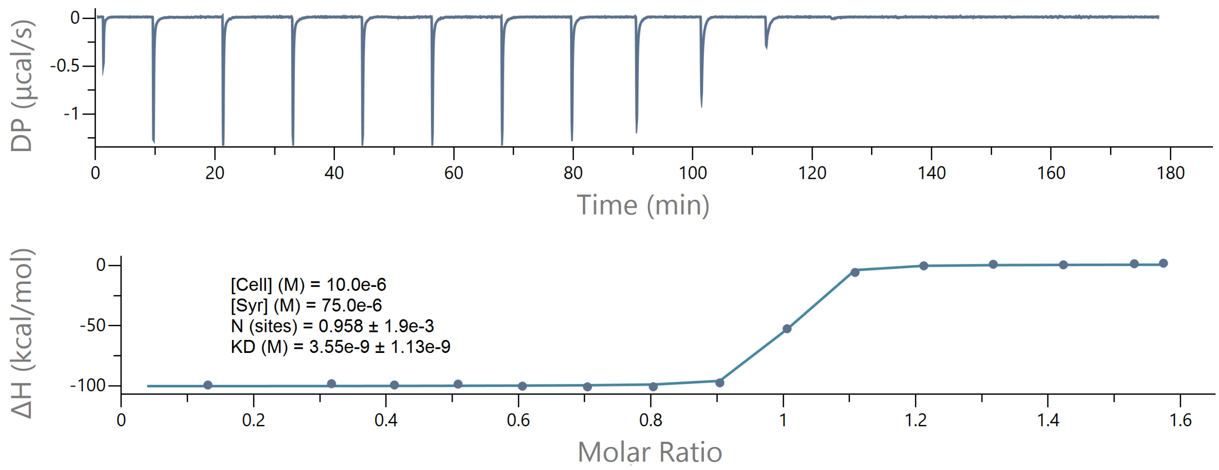

Figure S29. ITC experiment of PNA1 vs. model ssRNA_S2.

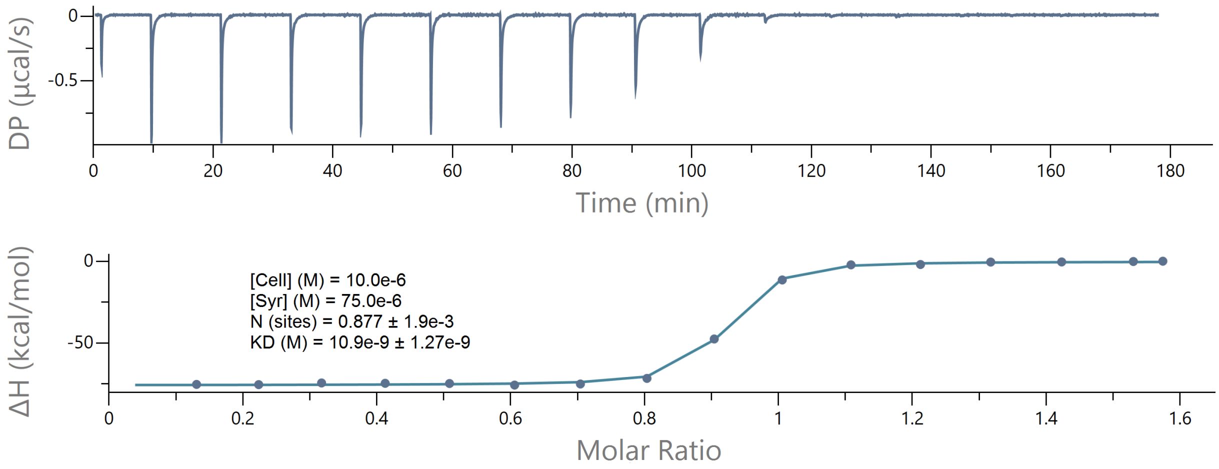

Figure S30. ITC experiment of PNA1m vs. model ssRNA_S2.

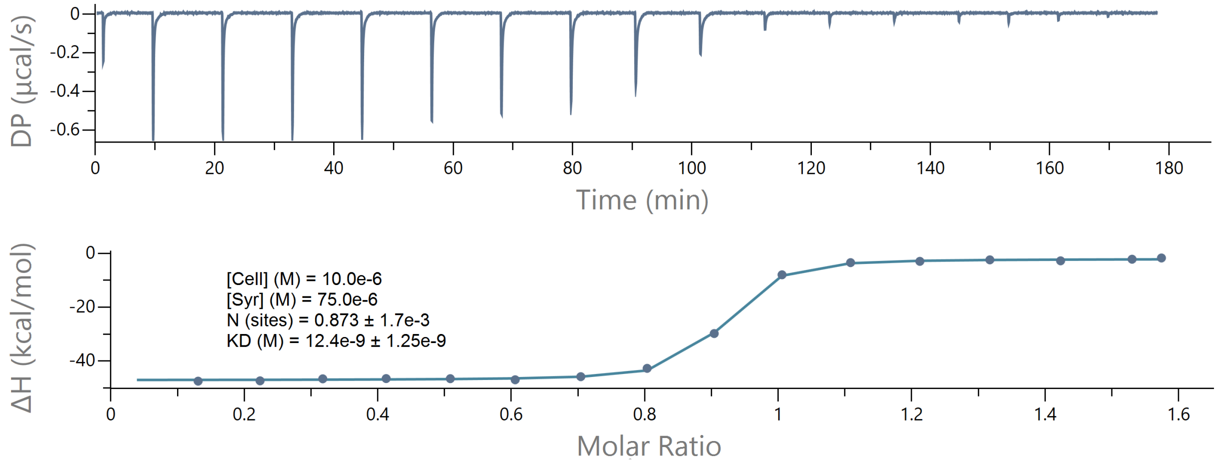

Figure S31. ITC experiment of PNA1d vs. model ssRNA_S2.

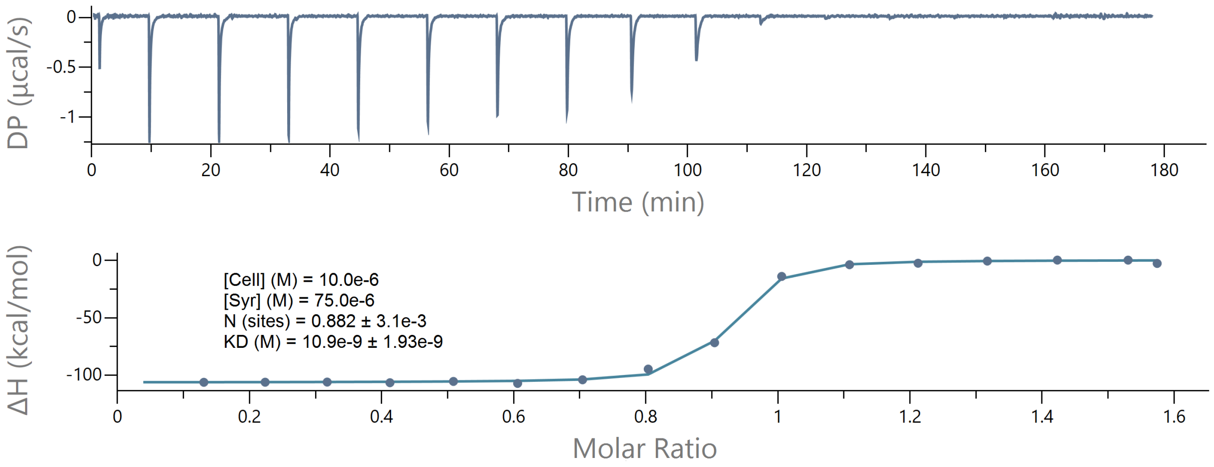

Figure S32. ITC experiment of PNA1d + PNA1t vs. model ssRNA_S2.

Table S3. PNA binding to stem 2 model ssRNA_S2

Name	Kd (M)	Ka (M^{-1})	ΔH(kcal/mol)	-TΔS(kcal/mol)	N
PNA1	3.6E-09	2.8E+08	-101	89.4	1.0
PNA1	3.7E-09	2.7E+08	-101	89.5	0.9
PNA1	3.5E-09	2.9E+08	-108	96.4	0.8
Average	3.6E-09	2.8E+08	-103	91.8	0.9
Std. Dev.	1.1E-10	8.6E+06	4.0	4.0	0.08
PNA1m	1.1E-08	8.8E+07	-73.2	62.4	1.0
PNA1m	1.1E-08	9.1E+07	-75.7	64.9	0.9
PNA1m	1.1E-08	8.9E+07	-83.1	72.2	0.8
Average	1.1E-08	9.0E+07	-77.3	66.5	0.9
Std. Dev.	2.5E-10	2.0E+06	5.2	5.1	0.08
PNA1d	1.2E-08	8.3E+07	-48.2	37.4	0.8
PNA1d	1.2E-08	8.1E+07	-44.9	34.2	0.9
PNA1d	1.2E-08	8.5E+07	-47.0	36.2	0.8
Average	1.2E-08	8.3E+07	-46.7	35.9	0.8
Std. Dev.	3.5E-10	2.4E+06	1.7	1.7	0.03
PNA1d + PNA1t	1.1E-08	8.9E+07	-95.1	84.3	0.8
PNA1d + PNA1t	1.1E-08	9.2E+07	-107	95.7	0.9
PNA1d + PNA1t	1.1E-08	9.2E+07	-105	94.0	0.9
Average	1.1E-08	9.1E+07	-102	91.3	0.8
Std. Dev.	1.7E-10	1.5E+06	6.4	6.2	0.06
